# Influence of Culture Conditions on Ex Vivo Expansion of T Lymphocytes and Their Function for Therapy: Current Insights and Open Questions

**DOI:** 10.3389/fbioe.2022.886637

**Published:** 2022-06-29

**Authors:** Harish Sudarsanam, Raymund Buhmann, Reinhard Henschler

**Affiliations:** Institute of Transfusion Medicine, University Hospital Leipzig, Leipzig, Germany

**Keywords:** T lymphocyte (T-cell), *ex vivo* expansion, CAR T cell, cell culture conditions, homing

## Abstract

*Ex vivo* expansion of T lymphocytes is a central process in the generation of cellular therapies targeted at tumors and other disease-relevant structures, which currently cannot be reached by established pharmaceuticals. The influence of culture conditions on T cell functions is, however, incompletely understood. In clinical applications of *ex vivo* expanded T cells, so far, a relatively classical standard cell culture methodology has been established. The expanded cells have been characterized in both preclinical models and clinical studies mainly using a therapeutic endpoint, for example antitumor response and cytotoxic function against cellular targets, whereas the influence of manipulations of T cells *ex vivo* including transduction and culture expansion has been studied to a much lesser detail, or in many contexts remains unknown. This includes the circulation behavior of expanded T cells after intravenous application, their intracellular metabolism and signal transduction, and their cytoskeletal (re)organization or their adhesion, migration, and subsequent intra-tissue differentiation. This review aims to provide an overview of established T cell expansion methodologies and address unanswered questions relating *in vivo* interaction of *ex vivo* expanded T cells for cellular therapy.

## Introduction

Adoptive T cell immunotherapy has evolved as a potent tool in numerous targeted therapies against tumors, autoimmune diseases, and other dysfunctions over the last 30 years. Donor lymphocyte infusions (DLIs) and virus-specific T cells are established allogeneic T cell therapies (Toprak 2018; Stroncek et al., 2019; Rath and Arber 2020; Waldman et al., 2020). Chimeric antigen receptor (CAR) T cell therapy is an advanced T cell therapy developed in the last decade and has strongly increased the clinical application of T cells (Ochi et al., 2010; Stroncek et al., 2019; Rath and Arber 2020; Waldman et al., 2020). In CAR T cell therapy, the T cells are genetically modified by transduction of a CAR transgene. The antigen-specific CAR construct is expressed on the surface of the T cells, which can directly identify the target antigens, for example on tumor cells. Currently, clinically established CAR T cell therapies involve mostly autologous T cells; however, there are ongoing developments toward “off the shelf” allogenic CAR T cell therapy (Feins et al., 2019; Stroncek et al., 2019; Depil et al., 2020).

The other known T cell therapies involve tumor-infiltrating lymphocytes (TILs) and T-cell receptor (TCR)–engineered T cells. TILs are tumor-reactive T cells isolated from patients and then expanded *ex vivo* in the presence of interleukin (IL)-2 before reinfusion into the patients. The role of TILs as therapeutic agents has been extensively studied in melanomas (Ochi et al., 2010; Ikeda, 2016; Dafni et al., 2019; Rath and Arber, 2020). TCR-engineered T cells are genetically engineered to express novel TCRs and target tumor antigens presented by major histocompatibility complex (MHC) (Ikeda, 2016; Met et al., 2019; Stroncek et al., 2019). Both TILs and TCR-engineered T cell therapies involve autologous T cells (Stroncek et al., 2019; Rath and Arber, 2020).

**FIGURE 1:**
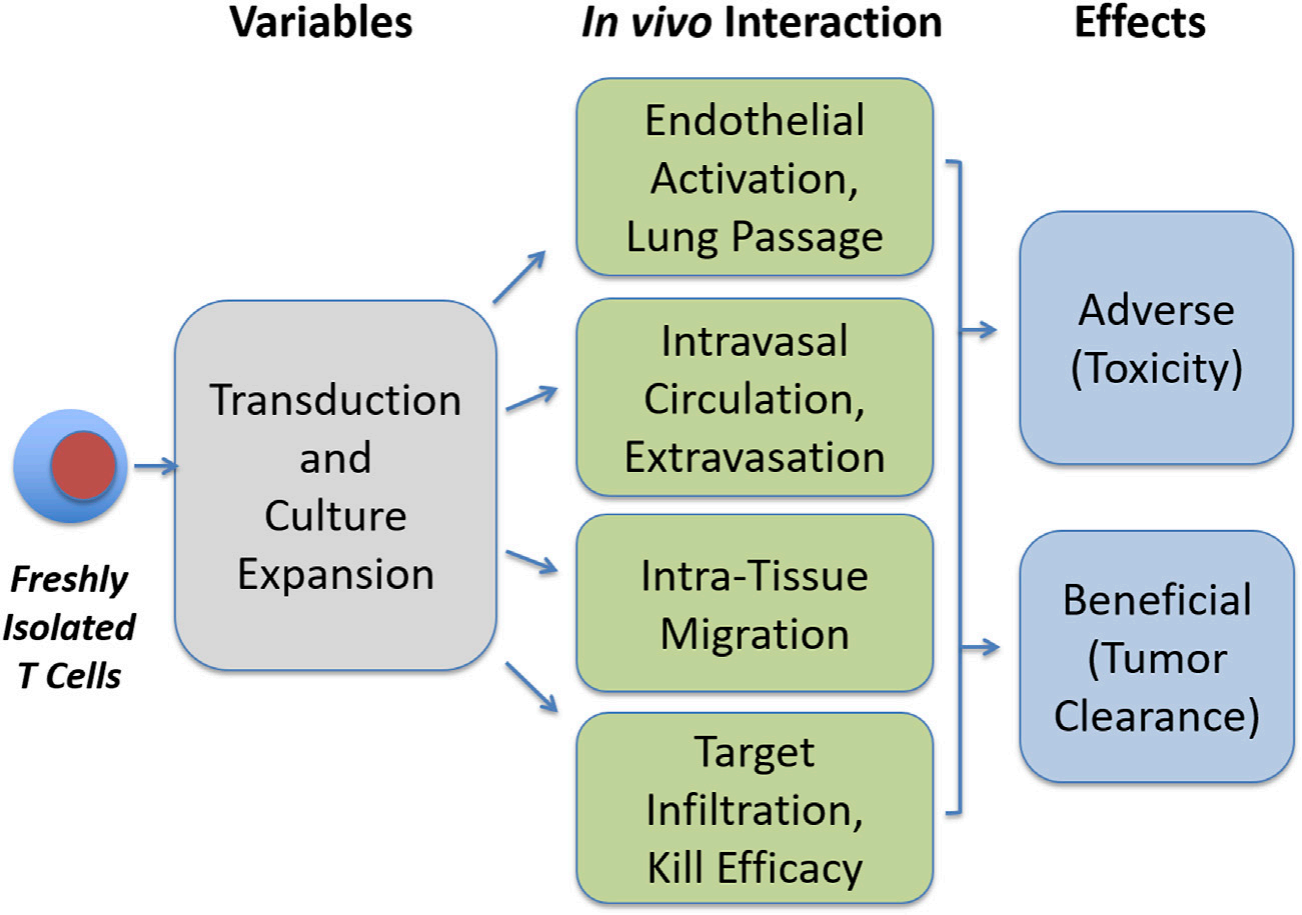
Open questions in T cell expansion research. Transduction and culture expansion may influence various aspects in the functionality of therapeutic T cells, which may in turn influence beneficial and adverse effects, as addressed in this review.

This review aims to summarize important developments in the aforementioned aspects of T cell therapy. We first give an overview of culture protocols, medium composition, serum supplementation, added cytokines, as well as for biotechnology approaches such as bioreactors required for *ex vivo* expansion. The second part of the review covers clinical studies with CAR T cells and explains not only the major benefits but also the toxicities observed. In the last part, we will review areas where important information is lacking. This includes the question of how transfused T cells are guided when they pass through the lung and while they circulate in the bloodstream, when they interact with endothelial cells to emigrate from blood, and when they migrate through tissues before they reach their targets and metabolic alterations. Figure 1 illustrates areas of research which warrant further exploration, that is, the identification of critical variables in culture protocols which affects metabolism, circulation behavior, migration, and target tissue infiltration of expanded T cells.

## Current Protocols and Future Perspectives in T cell Expansion

Generation of therapeutic T cell products requires several good manufacturing practice (GMP) steps including T cell isolation, activation of T cells, genetic modification (as required), *ex vivo* expansion, and quality control before reinfusion. T cell isolation may be divided into cell collection from leukapheresis or whole blood and subsequent cell enrichment by density gradient separation, counterflow elutriation, or antibody-based selection. T cells are then activated using CD3/CD28 antibodies, which may be coated on surface/beads or in soluble form. The next step of genetic modification may include either lentiviral or retroviral transduction, which are commonly used techniques for insertion of the CAR gene. The *ex vivo* expansion is a necessary step to obtain an adequate number of cells for infusion (Wang and Rivière, 2016; Stroncek et al., 2019; Abou-El-Enein et al., 2021).

**FIGURE 2:**
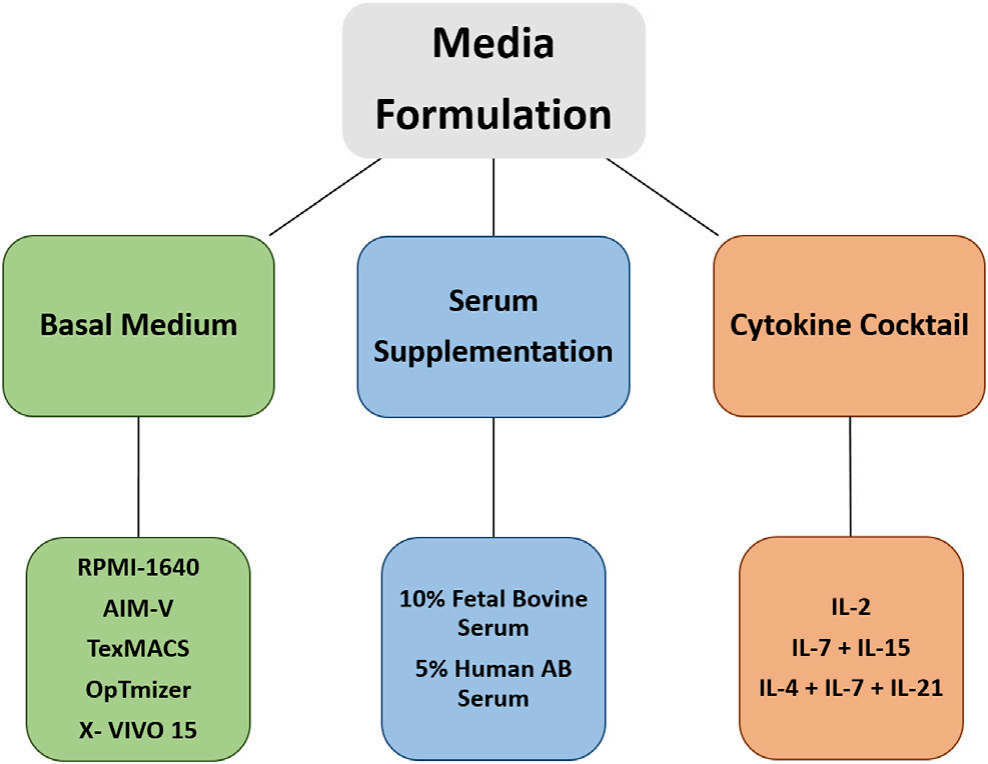
Currently used media formulations for *ex vivo* expansion of CAR T cells.

There is considerable variability in expansion protocols, which influences the antitumor activity and the associated toxicities. Hence, it is necessary to have efficient manufacturing technologies and standardized protocols in place (Barrett et al., 2015; Levine, 2015; Wang and Rivière, 2016; Stroncek et al., 2019; Abou-El-Enein et al., 2021). In the following section, we focus on current protocols and advanced technology concerning components in media formulations for *ex vivo* expansion of T cells. Figure 2 shows three main pillars of media formulation for CAR T cell expansion cultures.

### Basal Medium

The cell culture media formulation is a basic requirement for culturing of cells. Cell culture medium provides nutrients and growth factors and also essential constituents responsible for maintaining the osmolarity and pH buffering for optimal growth of the expanded cells outside their physiological environment (Henschler et al., 2018). Some of the basal culture media that have been successfully used for T cell expansion are RPMI, AIM-V, X-VIVO15, TexMACS, and OpTmizer T Cell Expansion media (Sato et al., 2009; Alnabhan et al., 2018; Medvec et al., 2018). According to a study presented by K. Sato et al. (2009), OpTmizer has an improved T cell expansion rate and effector functions compared to RPMI and AIM-V (Sato et al., 2009). Under identical culture conditions, the gene expression analysis of effector molecules and cytotoxicity functions of T cells were higher when cultured in OpTmizer than in AIM-V media, with the latter showing lower expression levels compared even to control PBMC (Sato et al., 2009). When comparing AIM-V and TexMACS, it was observed that TexMACS promotes the expansion of the T effector cell (T_eff_) phenotype, whereas AIM-V promoted the expansion of the T central memory cell (T_CM_). It was also observed that AIM-V favors IL-2 and IL-17 cytokine production and TexMACS assists in higher degranulation (CD107a) (Alnabhan et al., 2018). The T cell exhaustion markers TIM-3, PD-1, and LAG3 were analyzed in 5% human serum containing AIM-V medium and TexMACS medium. The median frequency of TIM-3 was observed to be more than 95%; similarly, PD-1 and LAG-3 were also upregulated but to a lesser extent when compared to TIM-3 (Alnabhan et al., 2018). It has been suggested that because of the lower activation status of T cells in AIM-V, this medium can be used for monitoring the T cell immune response (Sato et al., 2009). One of the advanced media formulations had been studied by Leney-Greene et al. (2020), Human Plasma like Medium (HPLM) can improve T cell activation and proliferation (Leney-Greene et al., 2020). The comparative analysis of activation markers CD69 and CD25 from five healthy donors indicates that HLPM supports higher T cell activation than RPMI, in both CD4 and CD8 T cells (Leney-Greene et al., 2020).

### Supplementation With Serum

Serum is an essential supplement in most cell cultures. Fetal bovine serum (FBS) has been the most commonly used serum supplement found in the literature (Festen et al., 2007; Brindley et al., 2012). However, pooled human serum (HS) from blood group AB donors is considered a preferred alternative for therapeutic purposes. Most CAR T cell expansion protocols contain a basal medium supplemented with either FBS or HS (Sato et al., 2009; Alnabhan et al., 2018; Medvec et al., 2018; Zhou et al., 2019). Irrespective of the medium used, the supplementation of serum has been investigated and found to improve T cell viability and proliferation when compared to expansion in serum-free media (SFM) (Sato et al., 2009; Alnabhan et al., 2018). The ratio of CD4/CD8 T cells was also found to be affected by the serum. In serum-containing TexMACS medium, the CD4/CD8 ratio was higher than that of serum-free TexMACS medium (Alnabhan et al., 2018). A chemically defined basal medium has been analyzed in T cell expansion and was shown to expand both naïve and memory T cells equivalently in either the presence or in the absence of 5% HS, whereas X- VIVO 15 could only expand naïve T cells in the presence or absence of serum but not memory T cells (Medvec et al., 2018). The CAR T cells manufactured from healthy donors and expanded in serum-containing media (SCM) showed transient tumor clearance, whereas SFM could generate more durable tumor control in the pre-B cell leukemic cell line NALM6, suggesting that serum affects the T cell function and *in vivo* persistence. However, this observation was attributed to the lot-to-lot variation in constituents of serum used (Medvec et al., 2018).

Despite FBS and HS being the most commonly used supplements in CAR T cell expansion, there is some concern regarding the usage of serum. FBS is a xenogeneic product, which increases the risk of immune reactions. HS is a preferred alternative, but as with FBS lacks consistency due to lot-to-lot variability. Serum of either source can also become one of the limiting factors in CAR T cell expansion during upscaling (Medvec et al., 2018). Different kinds of serum have also shown differences in transduction efficiency of the CAR gene for CAR T cell therapy, while there has been development of advanced SFM formulation for expansion of cells for therapeutic purposes (Medvec et al., 2018). The proliferation kinetics and specific phenotypical characteristics of T cells cultured in the aforementioned media do not match the growth of cells in a medium supplemented with serum (Brindley et al., 2012). There have been recent developments of serum alternatives, which can improve the growth kinetics and efficiency of expanded cells and also reduce the variability arising due to different pools of serum used. Human platelet lysate (hPL) and PhysiologixTM xeno-free (XF) hGFC (Phx) are known HS alternates (Torres Chavez et al., 2019a; Canestrari et al., 2019; Ghassemi et al., 2020). hPL is produced from transfusable human platelets. The addition of hPL has exhibited comparable expansion rates to serum-supplemented cultures but maintains comparably higher percentages of T_CM_. CAR T cells cultured with hPL demonstrates a superior antitumor effect in long-term *in vitro* coculture with NALM6 and Capan-1^PSCA^ (Torres Chavez et al., 2019a; Canestrari et al., 2019). The activation markers CD69 and CD25, as well as exhaustion makers TIM-3 and PD-1 on the surface of CAR T cells, had different expression levels when cultured with different serum supplements (FBS, HS, and hPL). It was observed that hPL promotes the expression of CD69 and CD25 on CAR T cells. While FBS supplementation had CAR T cells expressing the least levels of exhaustion markers, HS showed the highest expression of TIM-3, and hPL cultured cells had the highest expression of PD-1 (Torres Chavez et al., 2019a). XF-Phx is a human growth factor extracted from human whole blood fractions. A medium supplemented with XF-Phx could support a comparable expansion rate and enhanced transduction. The cytolytic capacity of CAR T cells has also improved upon culturing in XF-Phx–supplemented media (Ghassemi et al., 2020). OpTmizer supplemented with an alternative to both FBS and HS, OpTmizer T Cell Expansion supplement can support efficient T cell proliferation even in absence of serum (Sato et al., 2009; Smith et al., 2015).

### Addition of Cytokines

Cytokines are cell-signaling molecules crucial for activation, proliferation, and differentiation of cells and mimic similar functions in *ex vivo* cultures. The cytokine IL-2 was first discovered as a T cell growth factor and has pleiotropic functions in the life cycle of T cells (Smith, 1988; Ross and Cantrell, 2018). Activation of T cells leads to production of IL-2 and the expression of IL-2 receptors on its surface for autocrine signaling (Smith, 1988; Kim et al., 2006; Feau et al., 2011; Asao, 2014; Ross and Cantrell, 2018). Due to its pleiotropic functions including mediation of T cell survival and proliferation, IL-2 is the most frequently used cytokine in CAR T cell cultures (Besser et al., 2009; Luostarinen et al., 2017; Ghaffari et al., 2021).

IL-2 concentrations used in CAR T cell culture have a range between 10 and 7000 IU/ml (Huang et al., 2006; Besser et al., 2009; Luostarinen et al., 2017; Ghaffari et al., 2021). In a 14-day long culture of TIL generated from metastatic melanoma patients, the expansion rate of cells increased with increasing concentration of IL-2 only up to 600 IU/ml; cultures with a very high concentration of 1200 IU/ml were toxic to cells and cultures with no IL-2 cells underwent necrosis (Besser et al., 2009). IL-2 concentrations above 600 IU/ml also induced the expression of inhibitory markers CTLA-4 and CD33 (Besser et al., 2009; Ghaffari et al., 2021). A similar expansion curve was observed in T cells isolated from healthy donors, where T cells cultured with 50 IU/ml IL-2 had a higher expansion rate than cells cultured with 20 IU/ml of IL-2, but there was no difference at a higher concentration of 500 IU/ml of IL-2 (Ghaffari et al., 2021). It has been observed in multiple studies that the expansion rate of T cells is directly proportional to the concentration of IL-2, but only up to a certain range, above which the expansion rate reaches a plateau (Trickett et al., 2002; Huang et al., 2006; Besser et al., 2009; Luostarinen et al., 2017; Ghaffari et al., 2021). The cytotoxic activity and release of interferon (IFN)-γ of TIL also depend on the concentration of IL-2 in the cultures (Huang et al., 2006; Besser et al., 2009). There was a contrasting observation regarding the effect of IL-2 concentration on the proportion of CD4 and CD8 T cells, with Besser et al. (2009) observing no difference in the overall proportion, whereas Luostarinen, Kaartinen et al. (2017) observed that higher concentrations of IL-2 favor CD4 T cells (Besser et al., 2009; Luostarinen et al., 2017).

Successful T cell therapy requires both higher cytotoxic function and long-term survival upon infusion into patients. IL-2 favors the differentiation of naïve T cells to terminally differentiated T_eff_ with higher cytotoxic function and stronger response to chemokine-mediated homing to peripheral tissue. However, these T_eff_ cells are short-lived (Seder and Ahmed, 2003; Mahnke et al., 2013; Golubovskaya and Wu, 2016; Abramowski-Mock et al., 2017), whereas the intermediate differentiated states of stem cell memory T cells (T_SCM_) and T_CM_ are long-lived and have higher self-renewal capacity, thus showing longer persistence upon infusion when compared to the T_eff_ cells (Gattinoni et al., 2011; Mahnke et al., 2013; Golubovskaya and Wu, 2016; Luostarinen et al., 2017). Thus making the T_SCM_ and T_CM_ a promising differentiated T cell type for improved efficiency of CAR T cell therapy. Luostarinen, Kaartinen et al. (2017) observed that increasing the concentration of IL-2 led to a decrease in the early memory T cell (T_CM_, T_SCM_) and an increase in effector memory T cells (T_EM_) in the cultures. Cultures in 5 IU/ml of IL-2 had nearly 88% of early memory cells compared to only 61% in cultures with 300 IU/ml of IL-2 (Luostarinen et al., 2017). However, the T_SCM_ differentiation state showed significant variability with regards to culturing time, with some T cell cultures that did not show any delectable T_SCM_ cells on day 10, showed nearly 23% cells on day 20, but in cultures with 43% of T_SCM_ on day 10, had only 23% of T_SCM_ on day 20 (Luostarinen et al., 2017).

Apart from IL-2, other cytokines that are currently under investigation for application in CAR T cell cultures are IL-4, IL-7, IL-15, and IL-21. These cytokines are used in different concentrations and combinations to improve the CAR T cell viability, proliferation, and differentiation (Cha et al., 2010; Shi et al., 2013; Kravets et al., 2017; Ptáčková et al., 2018; Zhou et al., 2019; Marton et al., 2021). In the study presented by Zhou, Jin et al. (2019), it was observed that CAR T cells cultured in IL-7 + IL-15 have a higher proliferation rate and superior antitumor activity compared to cells cultured in IL-2-containing medium. The cultured CAR T cells also expressed higher levels of anti-apoptosis protein BCL-2 and lower expression of inhibitory molecule PD-1, exhibiting lower apoptosis rates and lower T cell exhaustion (Zhou et al., 2019).The addition of IL-7 + IL-15 to PBMC cultures enhanced the expansion rate of T cells even in the absence of an activation agent, explaining the agent-independent proliferation of T cells (Ramanathan et al., 2008; Ramanathan et al., 2009; Shi et al., 2013). A comparison between cord blood T cell cultures supplemented separately with IL-2 and IL-7 reveals that IL-7 promotes a higher CD4/CD8 ratio compared to cultures with IL-2 only; this may be due to the critical role of IL-7 in the survival of CD4^+^ memory T cells (Berglund et al., 2013). IL-7 + IL-15 also maintained high viability of T cells by suppressing the activation and inducing cell death (Mueller et al., 2003; Shi et al., 2013). The combination of IL-2 + IL-7 + IL-15 also increased the cytotoxic activity and release of IFN-γ by CD8^+^ T cells (Shi et al., 2013), but a contrasting result was observed in another study, with no significant difference in the cytotoxic activity or release of IFN-γ observed in cultures supplemented with IL-7 + IL-15 (Zhou et al., 2019). In T cell cultures, supplementing with IL-7 + IL-15 promotes the expansion of T_SCM_ and T_CM_ upon activation by anti-CD3/CD28 beads (Bere et al., 2010; Shi et al., 2013; Zhou et al., 2019). T cells cultured with IL-7 + IL-15 expressed higher levels of CCR7 and CXCR4 chemokine receptors and show enhanced migration toward the CCL21 gradient (Zhou et al., 2019). The study by Ptáčková et al. (2018), further analyzes a complex cytokine combination of IL-2 + IL-4 + IL-7 + IL-21 in CAR T cell cultures (Ptáčková et al., 2018). The cytokine cocktail of IL-4 + IL-7 + IL-21 promotes early memory cell types and prevents the expression of inhibitory receptors TIM-3 and PD-1 (Ptáčková et al., 2018). This indicates that the composition of cytokine cocktails has a significant effect on CAR T cell cultures, and hence it is essential to analyze cytokine cocktails in different concentrations and combinations before implementing them for culturing CAR T cells for clinical administration. Table 1 lists the commonly used media, serum supplement, and cytokine pool for the expansion of T cells. In summary, RPMI, AIM-V, and X-VIVO 15 are commonly used media for CAR T cell expansion with supplementation of either FBS or HS. In addition, IL-2 is one of the basic and commonly added cytokines to the CAR T cell cultures.

**TABLE 1:** Currently used media formulation for *ex vivo* expansion of CAR T cells.

CAR T Cell Media	Serum	Cytokines	References
RPMI-1640	10% FBS	100 U/ml IL-2	Johnson et al. (2015)
RPMI-1640 containing 45% Clicks medium	10% FBS	50 U/mL rh IL2	Watanabe et al. (2016)
RPMI-1640	10% FBS	50 U/mL of rh IL-2	Chicaybam et al. (2013)
50% AIM-V, 40% RPMI 1640	10% FBS	IL-2 (concentration not available)	Pinz et al. (2016)
45% Click’s media; 45% RPMI-1640	10% FBS	100 U/ml IL-2	Xu et al. (2016)
RPMI-1640 containing 45% Click’s medium	10% FBS	50 U/mL rh IL2	Bajgain et al. (2018)
50% RPMI-1640, 50% Click’s medium	10% FBS	50 U/mL rh IL2	Torres Chavez et al. (2019b)
45% RPMI-1640 and 45% Click’s medium	10% FBS	50 U/mL rIL-2	Caruana et al. (2015)
AIM V medium	5% FBS	40 U/mL & 300 U/mL rh IL-2	Long et al. (2015)
	5% HS	300 U/mL IL-2	Du et al. (2016)
	10% HS	100 U ⁄ ml rh IL-2	Teschner et al. (2011)
	5% HS	300 U/mL IL-2	Kim et al. (2015)
	10% FBS	20 ng/ml rh IL-2	Xu et al. (2019)
X- VIVO 15 medium	5% HS	200 U/ml IL-2	Eyquem et al. (2017)
		20 ng/ml IL-2	Valton et al. (2015)
		20 ng/ml rh IL-2	Galetto et al. (2014)
		30 U/mL rh IL-2 for CD4^+^ T cells	Wu et al. (2015)
		100 U/mL rh IL-2 for CD8^+^ T cells	

Abbreviation: FBS, fetal bovine serum; HS, human serum.

### Culture Period (Short-Term Versus Long-Term Expansion)

The culture period of CAR T cell expansion in most protocols is generally 7–14 days with few exceptions, which can therefore be considered a standard period (Lu et al., 2016; Coeshott et al., 2019; Pampusch et al., 2020). Longer culture periods have so far not been included in clinical studies. In this review, we therefore would not distinguish short- and long-term cultures. However, very short culture periods ranging from only a few hours to a few days have been described for IFN-γ–mediated enrichment of virus-specific T cells, for example cytomegalovirus (CMV)-specific T cells, which have been used in patients receiving stem cell transplantation (SCT). Here, the culture period is considered necessary mainly for the restimulation of the virus-specific T cells (Rauser et al., 2004; Feuchtinger et al., 2005; Feuchtinger et al., 2006; Tischer et al., 2014; Priesner et al., 2016; Kim et al., 2018). In a recent study, Ghassemi et al. (2022) developed a rapid protocol for CAR T cell manufacturing from nonactivated T cells within 24 h from the time of T cell collection. The CAR T cells generated through this rapid expansion protocol exhibited higher antitumor activity *in vivo* than CAR T cells manufactured through the standard protocol (Ghassemi et al., 2022).

### Manufacturing Technology

Standardization of activation reagent and media formulation has mostly been investigated in small-scale *in vitro* cultures. A single dose of expanded CAR T cells infused in patients is nearly 10^8^ to 10^9^ cells (Grigor et al., 2019; Abramson et al., 2020; Schuster et al., 2021; Westin et al., 2021). This makes efficient large-scale manufacturing technology under GMP conditions necessary for successful CAR T cell therapy. The earliest expansion technology was based on static culture conditions in a T flask or a culture bag. These systems were labor-intensive and required trained operators, which increases the possibility of contamination and variability in the quality and quantity of the cell product (Vormittag et al., 2018; Baudequin et al., 2021). There are currently multiple technologies available for upscaling the laboratory protocols to meet the required clinical dose.

The G-Rex system stands for the Gas Permeable Rapid Expansion system designed by WilsonWolf (Baudequin et al., 2021). The G-Rex system consists of a cell culture vessel/flask with a gas-permeable membrane at the bottom for ease of gas exchange. These flasks can be incubated in usual cell culture incubators. It is considered a practical and cost-effective way to scale up the CAR T cell cultures as it does not require additional specialized equipment or sensors (Baudequin et al., 2021; Garcia-Aponte et al., 2021; Westin et al., 2021). This system can be optimized to obtain a single therapeutic dose of CAR T cells per closed system. The G-Rex vessels are available with different surface areas ranging from 5 to 500 cm^2^ with a capacity of up to 4,500 ml. As this is a semi-automated technology, the cells are disturbed during sampling, which affects the cell expansion kinetics (Bajgain et al., 2014; Wang and Rivière, 2016; Ludwig et al., 2020; Baudequin et al., 2021; Garcia-Aponte et al., 2021).

The rocking motion (RM) bioreactors include the GE WAVE bioreactor system and Xuri cell expansion system (Wang and Rivière, 2016; Vormittag et al., 2018; Baudequin et al., 2021). These use sterile single-use cell bags for culturing CAR T Cells on a temperature-controlled rocking platform. The controlled rocking platform maintains bag inflation while providing sufficient mixing for improved gas exchange and homogeneity of nutrients required for cell expansion. They are available with sensors and controllers for perfusion, dissolved oxygen, and pH levels. The working volume depends on the size of the cell bag, which ranges from 2 to 50 L, and cell expansions can reach up to 1 × 10^7^ cells/ml. The major drawback of this technology is the possibility of mechanical and electrical failure of the rocking platform and controllers and contamination due to the semi-automated technology (Somerville et al., 2012; Janas et al., 2015; Wang and Rivière, 2016; Vormittag et al., 2018; Abou-El-Enein et al., 2021; Baudequin et al., 2021).

The aim is to develop efficient, highly controlled, and automated closed-system technologies, which can reduce labor-intensive procedures and eliminate any possibility of contamination (Abou-El-Enein et al., 2021; Baudequin et al., 2021). The CliniMACS Prodigy by Miltenyi Biotec and the Cocoon by Lonza are fully automated expansion technologies (Abou-El-Enein et al., 2021). The CliniMACS Prodigy is a one-system multistep technology, which integrates and automates the multistep process of CAR T cell manufacturing (Baudequin et al., 2021). This system performs cell separation, activation, lentiviral transduction, and expansion automatically in the single-use closed tubing system. The gas and media exchange and gentle mixing of cells are also automated with sensors and controllers (Apel et al., 2013; Mock et al., 2016; Baudequin et al., 2021; Garcia-Aponte et al., 2021). The CliniMACS Prodigy systems have been used for manufacturing CAR T cells as a substitute for the currently available technologies in clinical operation (Mock et al., 2016). One of the potential drawbacks of the CliniMACS Prodigy systems is that it is a single-component machine, where the culture vessel and centrifuge are the same. This leads to slower processing of samples as simultaneous processing of multiple batches is not possible with a single machine (Baudequin et al., 2021). In the report by Mock et al. (2016), the final composition of the product manufactured by CliniMACS Prodigy is similar to the product from the WAVE bioreactor. However, the yield with the CliniMACS Prodigy was 7.9 × 10^8^ ± 4.0 CAR + cells, whereas the yield in the WAVE bioreactor was 3.8 × 10^8^ ± 4.5 CAR19 + cells from a starting cell number of 0.7–1.2 × 10^8^ cells in both systems (Mock et al., 2016). In another study presented by Lock et al. (2017), with a starting material of 1 × 10^8^ T cells from DLBCL patients and healthy donors, the final product after 12 days of expansion in CliniMACS Prodigy contained 3.2 × 10^9^–4.9 × 10^9^ T cells from DLBCL patients and 2.9 × 10^9^–6.3 × 10^9^ from healthy donors (Lock et al., 2017).

Cocoon is a recently developed automated manufacturing system for T cell expansion by Lonza. This system uses a single-use cassette that is suitable for both suspension and adherent cells. This cocoon cassette performs magnetic separation, activation, transduction/transfection, and expansion of cells in a closed system, providing a high degree of standardization (Pharma.lonza, 2022). Similar to CliniMACS Prodigy, Cocoon integrates and automates both upstream and downstream processes of CAR T cell manufacturing (Titov et al., 2021). In multiple parallel process runs in the cocoon system, with both fresh and frozen cells, the cell yield was observed to be more than 10^9^ cells with more than 95% purity and more than 97% viability of T cells, showing a high reproducibility through a standardized workflow (Pharma, 2021).

Taken together, culture conditions have a major impact on the final CAR T cell product obtained for transfusion. The identification of optimal CAR T cell expansion conditions is complex. The contrasting results in different comparative studies focusing on a particular expansion condition may be due to differences in other variables that affect the culture conditions. However, now a number of industrially established CAR T cell therapies are available against hematological tumors, which have shown promising results.

### Establishment of Clinical CAR T Cell Therapies

In 1989, Eshhar et al. for the first time described the principle of expressing a specifically designed transgene encoding a chimeric transmembrane antigen receptor (the CAR) in autologous T lymphocytes which can recognize and bind a therapeutically relevant (tumor) cell antigen triggering the activation of the T cells in order to eliminate target cells (Gross et al., 1989). The clinical breakthrough of this technology has become evident 2 decades later by the achievement of high cure rates in children with acute lymphatic leukemia by anti-CD19 CAR T cells by the group Carl June (Kalos et al., 2011). As more clinical applications were developed, the complexity of the isolation, culture, genetic engineering, and the cell expansion steps under GMP conditions prompted the pharmaceutical industry to engage in the establishment of specific product lines (Abou-El-Enein et al., 2021; Alnabhan et al., 2018; Sato et al., 2009; Zhou et al., 2019; Smith et al., 2015; Westin et al., 2021; Bajgain et al., 2014; Somerville et al., 2012; Elavia et al., 2019). Table 2 gives several CAR T cell products which have entered large-scale clinical production as study medications. Differences in cell dosage, as well as the number of patients treated, and clinical outcomes have been reported in these studies. However, information regarding the long-term persistence and killing efficiency have received less attention. Globally, currently, a limited number of large production facilities exist for the manufacturing of these cell therapeutics. In addition to globally acting pharmaceutical companies, smaller-scale trials with locally manufactured CAR T cell products are underway and have led to important progress in therapy and contain indications such as HIV, cancer, and autoimmune diseases which are reviewed elsewhere (Clinicaltrials, 2022).

**TABLE 2:** Commercially produced CAR T cell therapies and initial treatment indications.

References	O'Leary et al. (2019)	Bouchkouj et al. (2019)	Schuster et al. (2021)	Wang et al. (2020)	Shah et al. (2021)	Abramson et al. (2020)	Bouchkouj et al. (2022)	Sharma et al. (2022)	Berdeja et al. (2021)
Clinical outcome	CR 63%	CR 51%	CR 39%	CR 67%	CR 56%	CR 53%	CR 60%	sCR 28% VGPR 25%	sCR 67% VGPR 26%
		PR 21%	PR 14%	PR 27%	CRi 15%	PR 20%	PR 31%	PR 19%	PR 4%
Number of patients	63	101	115	68	55	269	120	100	97
Dosage (CAR-positive viable T cells)	0.2 to 5.0 × 10^6^ cells/kg body weight	2.0 × 10^6^ cells/kg body weight	0.1 × 10^8^ to 6 × 10^8^ cells (flat dose)	2.0 × 10^6^ cells/kg body weight	1.0 × 10^6^ cells/kg body weight	4.4 × 10^8^ to 15.6 × 10^8^ cells (multiple doses)	2.0 × 106 cells/kg body weight	3 × 10^8^ to 4.6 × 10^8^ cells (flat dose)	0.5 to 1.0 × 10^6^ cells/kg body weight
Disease type and state	R/R acute Lymphoblastic leukemia	R/R large B cell lymphoma	R/R Diffuse large B cell lymphoma	Mantle cell lymphoma	R/R acute Lymphoblastic leukemia	R/R large B cell lymphoma	R/R Follicular lymphoma	R/R multiple myeloma	R/R multiple myeloma
Target antigen	CD19	CD19	CD19	CD19		CD19	CD19	BCMA	BCMA
Manufacturer	Novartis	Kite Pharma	Novartis	Kite Pharma		Juno Therapeutics	Kite Pharma	Celgene Corporation	Janssen Biotech
Product	*tisagenlecleucel*	*axicabtagene ciloleucel*	*tisagenlecleucel*	*brexucabtagene autoleucel*		*lisocabtagene maraleucel*	*axicabtagene ciloleucel*	*Idecabtagene vicleucel*	ciltacabtagene autoleucel
Date of approval	2017	2017	2018	2020		2021	2021	2021	2022

Abbreviation: R/R, relapsed or refractory; CR, complete remission; PR, partial remission; CRi, complete remission with incomplete hematological recovery; sCR, stringent complete response rate; VGPR, very good partial response

According to the European Medicines Agency’s Committee for Advanced Therapies, CAR T cells are an advanced therapy medicinal product (ATMP) (Buechner et al., 2018; Vucinic et al., 2021). Industrial production of CAR T cells requires standardization of multi-process manufacturing steps in order to reduce the variability of the final product and meet the efficacy observed in clinical studies (Wang and Rivière, 2016; Stroncek et al., 2019; Abou-El-Enein et al., 2021). Hence, GMP protocols with automated manufacturing systems and trained personnel are essential (Gee, 2018). Standardization of culture medium and supplements can be achieved by using a clinical-grade serum-free, chemically defined medium from the same vendor for *ex vivo* expansion of CAR T cells. Although an essential supplement for CAR T cell expansion, serum induces variability in the composition of the final product (Medvec et al., 2018). The upscaling of CAR T cell products from bench to bedside requires closed-system manufacturing technologies, and automation is favorable but not essential for current working standards (Gee, 2018). As discussed above, CliniMACS Prodigy is an automated manufacturing system currently used for manufacturing CAR T cells. Another automated manufacturing system is Cocoon by Lonza (Baudequin et al., 2021; Pharma.lonza, 2022). A further step toward standardized manufacturing is centralized-manufacturing units. As observed in the case of CAR T cell product, for example by Novartis, the manufacturer of Kymriah, there are only six manufacturing units, three in Europe and two in United States; and one recently approved in Japan (Novartis, 2021). Similarly, in the case of Bristol Myers Squibb, the two approved CAR T cell products are manufactured in five units (Bms, 2021). Validation and qualification of all cell therapy products before infusion is part of GMP protocol. Finally, trained personnel and management of medical records is also essential for Standardized CAR T cell product (Gee, 2018; Fritsche et al., 2020).

Currently, all the six clinically approved CAR T cell therapies use second-generation CAR construct but with different co-stimulatory domains (Meng et al., 2021). A CAR structure can be divided into four major domains, an extracellular antigen binding domain, a spacer domain, a transmembrane domain, and a cytoplasmic domain. First-generation CARs contain only the CD3ζ signaling domain, whereas in second-generation CARs, an additional co-stimulatory endodomain is added to the signaling domain. The third-generation CARs have multiple co-stimulatory endodomain (Holzinger and Abken, 2019; Huang et al., 2020; Jayaraman et al., 2020).

T cells redirected for antigen-unrestricted cytokine-initiated killing (TRUCKs), the fourth-generation CARs have additional signal for inducible cytokine release (Chmielewski and Abken, 2015; Chmielewski and Abken, 2020; Dragon et al., 2020). The inducible cytokines studies with TRUCKs are IL-2, IL-7, IL-12, IL-15, IL-17, and IL-21 (Tian et al., 2020). The release of these cytokines improves the antitumor activity, longer persistence, and resistance to inhibitory signals (Petersen and Krenciute, 2019). These cytokines are deposited in tissues and induce a secondary immune response that is otherwise inaccessible by CAR T cells in solid tumors (Chmielewski and Abken, 2015). Furthermore, there has been the development of universal CAR (UniCAR) T cell systems and chimeric autoantigen receptor (CAAR) T cells (Holzinger and Abken, 2019). The uniCAR system is divided into two functional components. The CAR transgenes inserted in T cells are similar to the second generation of the CAR construct, except that the extracellular antigen-binding domain now does not recognize tumor antigen but recognizes an epitope present on target modules (TMs). TMs are short-lived molecules with an epitope and a specific tumor antigen-binding domain. As UniCAR systems are controlled by TM, their efficiency and associated toxicities can be managed by titration of TM (Cartellieri et al., 2015; Cartellieri et al., 2016; Albert et al., 2017; Feldmann et al., 2017; Bachmann et al., 2018; Loureiro et al., 2018; Bachmann, 2019; Jureczek et al., 2019; Arndt et al., 2020; Lin et al., 2021). CAAR T cell therapy is directed toward autoimmunity, more specifically toward B cell-mediated autoimmune disorders. CAAR T cell construct is also similar to the second-generation CAR construct, except that the ectodomain is now a specific antigen instead of a ligand binding site. The antigen presented by the CAAR T cell is an autoantigen targeted by autoantibody produced by B cells during autoimmune disorders. The binding of autoantigens presented by CAAR to the autoantibodies leads to activation of CAAR T cells and eliminates auto-reactive B cells (Ellebrecht et al., 2016; Meng et al., 2018; Ellebrecht et al., 2019; Sadeqi Nezhad et al., 2020; Aghajanian et al., 2022).

## Toxicities Associated with CAR T Cell Therapy

### Cytokine Release Syndrome

CRS is one of the most common toxicities in CAR T cell therapy. CRS is a systemic inflammatory response, observed due to hypersecretion of cytokines and other inflammatory markers during the CAR T cell–mediated tumor clearance mechanism (Frey and Porter, 2019; Murthy et al., 2019). CRS is a result of a complex interplay between T cells, myeloid cells, endothelial cells, tumor cells and the signals released by them. Its initiation upon early contacts with host immune cells and endothelial cells has been discussed in the later sections.

### Mediators of Cytokine Release Syndrome

The activation of infused CAR T cells and interaction with tumor cells leads to the release of several cytokines including IL-2, soluble IL-2Rα, IFNγ, IL-6, tumor necrosis factor (TNF)-α, soluble IL-6R, and GM-CSF. Activation of monocytes and macrophages by these factors, in turn, leads to the secretion of IL-1, IL-1RA, IL-10, IL-6, soluble IL-6R, IL-8, IL-10, IL-12, TNF-α, CXCL10 (IP-10), CXCL9 (MIG), IFNα, CCL3 (MIP-1α), CCL4 (MIP-1β), and MCP1, and further, the activation of endothelial cells release IL-6 and IL-8. Other inflammatory mediators released include IL-1, IL-18, MCP1, ferritin and C-reactive protein (CRP), and inducible nitric oxide synthase (iNOS) (Shimabukuro-Vornhagen et al., 2018a; Wang and Han, 2018a; Neelapu et al., 2018; Garcia Borrega et al., 2019; Murthy et al., 2019; Yáñez et al., 2019). Most of the cytokines released then initiate a feed-forward loop, resulting in cytokine hypersecretion and CRS (Wang and Han, 2018b).

IL-6, IFNγ, and TNF-α are cytokines released early in CRS from activated CAR T cells (Rose-John, 2012). These cytokines are consistently found in CRS and play a major role in its toxicity. IL-6 is released by different immune cells (T cells, monocytes/macrophages, dendritic cells) and by endothelial cells upon activation. IL-6 displays both pro-and anti-inflammatory roles. IL-6 signaling occurs through two different pathways. In “classical signaling”, IL-6 binds to the IL-6 receptor (IL6R) present on the cell surface; in the case of “trans-signaling” the IL-6 binds to soluble IL-6 receptors (sIL-R). IL-6/IL-6R binds to gp130 and initiates signaling through its intracellular domain *via* the JAK/STAT pathway (Rose-John et al., 2006; Rose-John, 2012). IFN-γ and TNF-α are released by immune effector cells such as activated CAR T cells and by lysis of tumor cells and induce activation of other immune cells and endothelial cells (Cosenza et al., 2021). Macrophages are activated by IFN-γ which leads to hypersecretion of IL-6, IL-10, and TNF-α (Tau and Rothman, 1999). TNF-α increases vascular permeability (Parameswaran and Patial, 2010). IFN-γ and TNF-α have also been associated with impairment of the blood–brain barrier (BBB) and activation of microglia cells in CAR T cell therapy associated with neurotoxicity (Wang and Han, 2018b; Murthy et al., 2019).

### Therapeutic Agents Against Cytokine Release Syndrome

As IL-6/IL-6R is one the major driving factors of CRS, blockade of IL-6/IL-6R and its signaling pathway is considered for clinical management of CRS. Tocilizumab is an FDA-approved anti–IL-6R antibody, which binds to both membrane-bound and soluble IL-6R. Siltuximab and clazakizumab are monoclonal antibodies targeting IL-6 (Shimabukuro-Vornhagen et al., 2018a; Kang et al., 2019; Murthy et al., 2019). Ruxolitinib blocks the downstream signaling induced by gp130 by inhibiting the JAK/STAT pathway (Kenderian et al., 2017; Murthy et al., 2019). Lenzilumab is an anti-GM-CSF antibody for neutralizing human GM-CSF produced during CRS. It does affect the efficacy of CAR T cells, rather enhancing its activity. It is currently in clinical trials for axicabtagene ciloleucel CAR T cell therapy (Sterner et al., 2019; Siegler and Kenderian, 2020).

Etanercept is a soluble form of TNF-α receptor administered in case of severe CRS. It has shown promising results in rapidly resolving pediatric patients but not in adult patients. Etanercept is administered alone or in combination with tocilizumab (Zhang et al., 2018; Siegler and Kenderian, 2020). Anakinra is an IL-1 receptor antagonist and blocks IL-1-mediated CRS. IL-1 release is preceded by the release of IL-6 and administration of anakinra controls CRS and neurotoxicity. It is also used for treating patients with hemophagocytic lymphohistiocytosis (HLH). It also downregulates the iNOS produced by macrophages (Norelli et al., 2018; Siegler and Kenderian, 2020). Table 3 lists antibodies used in CRS and its targets. In summary, CRS is a major complication in CAR T cell therapy and can be counteracted by specific neutralizing antibodies. The influence of CAR T cell expansion culture conditions on the occurrence of CRS is not known.

**TABLE 3:** Therapeutic agents against the targets that play a major role in CRS.

Therapeutic Agents	Targets	Advantages	Disadvantages	References
Tocilizumab	IL-6 receptor	1) Direct anti-cytokine drug in the treatment of CAR-T-associated CRS; 2) Does not affect the efficacy of CAR-T-cells; 3) Very well-tolerated drug with minimal adverse events	1) cannot be used in the management of ICANS; 2) Poor penetration of the blood–brain barrier	Si and Teachey. (2020); Siegler and Kenderian. (2020)
Siltuximab	IL-6	1) prevents binding of IL-6 to both soluble and membrane-bound IL-6R; 2) prevents the exposure of IL-6 to the central nervous system by blocking IL-6 instead of IL-6R	None identified	Shimabukuro-Vornhagen et al. (2018a); Kang et al. (2019); Murthy et al. (2019)
Ruxolitinib	JAK/STAT signaling pathway	1) Diminished inflammatory cytokines such as IFN-γ & TNF-α, and alleviated symptoms of CRS.; 2) Prolonged overall survival in a mouse model of CAR T cell–induced CRS; 3) has been used to treat steroid-refractory CRS and severe CRS patients; 4) in clinical trials	suppress CAR-T-cell cytotoxicity *in vitro*	Kenderian et al. (2017); Murthy et al. (2019); Dufva et al. (2020); Li et al. (2021); Pan et al. (2021); Zi et al. (2021); Xu et al. (2022)
Lenzilumab	GM-CSF	1) GM-CSF inhibition by lenzilumab enhances CART19 cell proliferation, antitumor activity, and overall survival in patient-derived xenograft model; 2) Prevents the development of CRS and significantly reduces the severity of neurotoxicity; 3) clinical study: ZUMA-19	None identified	(Sterner et al., 2019; Siegler and Kenderian, 2020)
Etanercept	TNF-α	1) Successfully cured CRS in patients with elevated levels of TNF-α; 2) CAR T cell proliferation and effector functions were not affected; 3) in clinical trials	None identified	Grupp et al. (2013); Zhang et al. (2018); Siegler and Kenderian. (2020); Zhang et al. (2021); Zhou et al. (2021)
Anakinra	IL-1R	1) Standard treatment for the management of steroid-refractory ICANS with or without CRS; 2) equally effective in preventing CRS mortality as compared to tocilizumab; 3) has been effective in treating patients with HLH.	None identified	Norelli et al. (2018); Siegler and Kenderian. (2020); Wehrli et al. (2022)

Abbreviation: IL-6, (interleukin) 6; IFN-γ, (Interferon) gamma; GM-CSF, granulocyte-macrophage colony stimulating; TNF-α, tumor necrosis factor-alpha; HLH, hemophagocytic lymphohistiocytosis.

### Grading System for Cytokine Release Syndrome

Although there are multiple studies on CAR T cell therapy, the severity of toxicities between different studies is usually not directly compared, mainly since there are multiple grading systems used in different study trials to determine the severity of CRS and its related toxicities. Three commonly used grading systems for CRS and other toxicities are the Penn Scale, developed by the University of Pennsylvania; NIH consensus criteria also called as Lee scale; and the ASTCT scale developed by the American Society for Transplantation and Cellular Therapy (ASTCT) (Lee et al., 2014; Porter et al., 2018; Lee et al., 2019; Schuster et al., 2020).

### Immune Effector Cell Associated Neurotoxicity Syndrome (ICANS)

ICANS was also referred to as CAR T cell–related encephalopathy syndrome (CRES) until the publication of a new grading system by ASTCT (Lee et al., 2019). ICANS is one of the most common CAR T cell therapy–related toxicities associated with CRS. Data from multiple clinical trials data indicate that ICANS may be associated with the CAR construct and the antigen targeted by CAR T cells also, and the severity of ICANS can be correlated with the severity of CRS, higher tumor burden, a higher dose of CAR T cells, and their higher *in vivo* expansion rates (Turtle et al., 2017; Brudno et al., 2018; Gauthier and Turtle, 2018; Park et al., 2018; Santomasso et al., 2018; Garcia Borrega et al., 2019). It has been observed in the ZUMA-1 CAR T cells trial that the onset of ICANS occurs later than CRS and that symptoms persist longer than CRS symptoms (Santomasso et al., 2018). Symptoms of ICANS range from mild headaches, fatigue, and mild aphasia to severe manifestation in the form of seizures, raised intracranial pressure with cerebral edema, and coma (Santomasso et al., 2018; Garcia Borrega et al., 2019).

ICANS is associated with increased permeability of the BBB, vascular and endothelial dysfunctions. The cytokines and other immune mediators are released during CRS, which lead to systemic inflammation also correlated with the severity of ICANS (Gust et al., 2017; Turtle et al., 2017; Santomasso et al., 2018). The role of IL-1 and GM-CSF released by monocytes has been studied in a xenograft mouse model, where blockade of IL-1 and GM-CSF resulted in significant reduction in ICANS (Sterner et al., 2019). Increased permeability of the BBB, higher CSF/blood ratio of cytokines, and high numbers of CAR T cells in CSF have been observed in case of severe ICANS (Gauthier and Turtle, 2018).

Preclinical studies indicated that blockade of IL-1 and GM-CSF by anakinra and lenzilumab can be an approach toward the management of ICANS. However, it should be noted that IL-6 blockade by tocilizumab used in case CRS is not effective in case of ICANS (Shimabukuro-Vornhagen et al., 2018a; Norelli et al., 2018; Garcia Borrega et al., 2019; Yáñez et al., 2019). Table 4 provides details of common toxicities associated with CAR T cell therapies as observed in major clinical studies.

**TABLE 4:** CAR T cell–associated toxicities in major clinical studies.

References		O'Leary et al. (2019)		Bouchkouj et al. (2019)	Schuster et al. (2021)	Wang et al. (2020)	Shah et al. (2021)	Abramson et al. (2020)	Bouchkouj et al. (2022)	Sharma et al. (2022)	Berdeja et al. (2021)
ICANS	grade ≥3	18%		31%	11%	31%	25%	10%	21%	4%	9%
	Any grade	65%		87%	20%	63%	60%	30%	77%	28%	21%
CRS	grade ≥3	49%		13%	23%	15%	24%	2%	8%	9%	4%
	Any grade	79%		94%	57%	91%	89%	42%	84%	85%	95%
Number of patients		63		101	115	68	55	269	120	100	97
Clinical studies		ELIANA		ZUMA-1	JULIET	ZUMA-2	ZUMA-3	TRANSCEND	ZUMA-5	KarMMa	CARTITUDE-1
Disease type and state		R/R acute Lymphoblastic leukemia		R/R large B cell lymphoma	R/R diffuse large B cell lymphoma	R/R Mantle cell lymphoma	R/R acute Lymphoblastic leukemia	R/R large B cell lymphoma	R/R Follicular lymphoma	R/R multiple myeloma	R/R multiple myeloma
Product		tisagenlecleucel	axicabtagene ciloleucel		*tisagenlecleucel*	*brexucabtagene autoleucel*		*lisocabtagene maraleucel*	*axicabtagene ciloleucel*	*Idecabtagene vicleucel*	ciltacabtagene autoleucel

### Strategies to Improve CAR T Cell Therapy and Underexplored Areas of Research

Tumor burden is one of the factors involved in the toxicity; high tumor burden at the start of the therapy increases the risk of CAR T cell–associated toxicity. In most cases of CRS, severe toxicity is observed after the administration of the first dose. High tumor burden is one of the important predictors of the severity of CRS (Brentjens et al., 2013; Turtle et al., 2016a; Shimabukuro-Vornhagen et al., 2018b). Hence, a bridging therapy after apheresis and before CAR T cell infusion can help reduce the tumor burden. Radiation therapy is a widely considered bridging therapy to control rapid disease progression and reduce tumor burden until CAR T cell infusion (Sim et al., 2019; Pinnix et al., 2020; Leick et al., 2021; Deshpande et al., 2022). A high CAR T cell dose not only helps in clearing the tumor but also increases the increase of toxicity; this can be countered by multiple smaller doses of CAR T cells (Turtle et al., 2016a; Gust et al., 2017; Wang and Han, 2018b). In multiple dose escalation studies, the infused CAR T cell dose ranges from 10^5^ cells/kg to 10^8^ cells/kg, and the optimal cell dose was found to be close to 10^6^ cells/kg, whereas a cell dose higher than 10^7^ cells/kg was associated with severe toxicity (Lee et al., 2015; Turtle et al., 2016a; Turtle et al., 2016b; Park et al., 2016; Gust et al., 2017; Wang and Han, 2018b; Fry et al., 2018; Dasyam et al., 2020; Siddiqi et al., 2022). The “on-target off-tumor effect” leads to long term B cell aplasia in CD19 CAR T cell therapy, which can be countered by a uniCAR T cell system and by “switch-on/switch-off” CAR T cells (Holzinger and Abken, 2019; Yáñez et al., 2019; Lin et al., 2021). The presence of existing neurological comorbidities in patients undergoing CAR T cell therapy also increases the risk of ICANS (Gust et al., 2017). Thus, a stringent and clear criterion for participation is necessary.

The occurrence of CRS is more profound in second-generation CAR constructs with additional co-stimulatory signaling domain than first-generation constructs, where only the CD3 signaling domain was present. In second-generation CAR constructs, the CD28 co-stimulatory domain has higher CRS incidence than the 4-1BB CARs co-stimulatory domain, and this shifts the focus on advanced CAR constructs, which have been described earlier. (Savoldo et al., 2011; van der Stegen et al., 2015; Neelapu et al., 2017; Shimabukuro-Vornhagen et al., 2018b). Also, CARs targeting inhibitory ligands and TRUCKs with inducible cytokine transgene improves CAR T cell functions and prevents T cell exhaustion in solid tumors (Holzinger and Abken, 2019).

### Endothelial Cell Activation by CAR T Cells

Endothelial cells maintain the blood fluidity and blood flow and transmembrane permeability, and they control the activation and adhesion of circulating leukocytes through various mechanisms. Failure of endothelial cells to perform any of these functions can be termed endothelial cell dysfunction (Pober and Sessa, 2007). The activation of endothelial cells from the quiescent stage to the activated stage is an inflammatory response and can be induced through two different mechanisms. Type I endothelial activation is a rapid response through ligand binding to GPCR receptors such as histamine H1 receptors (Pober and Cotran, 1990), whereas type II activation is a much slower but sustained response mediated by TNF-α and IL-1 through transcriptional changes in endothelial cells (Pober and Cotran, 1990; Pober and Sessa, 2007). The release of TNF-α and IL-1 during CRS has already been reviewed in the earlier section.

Endothelial cell activation is one of the CAR T cell therapy–associated toxicity (Gavriilaki et al., 2020). Persistent activation of endothelial cells CRS during CAR T cell therapy has also been described to result in endothelial dysfunction (Page and Liles, 2013; Gust et al., 2017). The concentration of angiopoietin2 (ANG2), von Willebrand factor (VWF), and IL-8, which are stored in Weibel–Palade bodies and released upon endothelial activation, was found to be higher in patients after CAR T cell infusion (Gust et al., 2017; Hong et al., 2021). There was also an increase in the ANG2:ANG1 ratio (Gust et al., 2017). A comparison in patients with mild and severe CRS in the first month after CAR T cell infusion against B-ALL showed that endothelial cell activation correlates with the severity of CRS (Hong et al., 2021). The endothelial markers were also found to be at higher levels during the peak of CRS compared to the initial or recovery phase of CRS, showing a direct association with the rise and decline of CRS (Hong et al., 2021). Similarly, greater levels of the aforementioned endothelial activation markers were observed in patients with severe neurotoxicity within 1 week of receiving CAR T cell therapy (Gust et al., 2017). The VWF concentration in serum was four to five fold higher in patients with severe neurotoxicity (grade ≥4 neurotoxicity) than in serum from healthy donors (Gust et al., 2017). The soluble form of cell adhesion molecules such as E-selectin, VCAM1, and ICAM1 was also found to be elevated in patients with severe CRS (Hong et al., 2021). The endothelial activation markers were also high in patients with renal and hepatic dysfunction due to CRS following CAR T cell therapy.

Endothelial activation markers can predict the severity of CRS and neurotoxicity in undergoing CAR T cell therapy. Currently, CAR T cell toxicity is monitored based on the rise in body temperature for initial 36 hrs after infusion of CAR T cells (Gust et al., 2017). Decision tree models have been developed based on endothelial activation markers to predict the occurrence of severe CRS. Endothelial activation markers peaked earlier than markers for CRS after CAR T cell infusion. Markers such as sVCAM1, sICAM1, and ANG2:ANG1 ratios were able to predict the severity of CRS based on modeling (Hong et al., 2021). In patients with severe neurotoxicity, the ANG2:ANG1 ratio was higher even before the beginning of preconditioning of patients through chemotherapy-based lymphodepletion. The severity of neurotoxicity also correlated with an increase in ANG2 levels within 24 hrs of CAR T cell infusion, preceding the onset of other toxicities. Thus, CAR T cell infusion is associated in endothelial cell activation, unlike after transfusion of un-manipulated T cells. The influence of *ex vivo* culture on endothelial cell activation after CAR T cell infusion needs further investigation.

### Efficiency and Functionality of CAR T Cell Therapies With Regard to Their Migration Capabilities

The capacity of expanded T cells to affect their target (e.g. killing of tumor cells) has naturally been the primary focus of current scientific attention and reports in the literature (Roschewski et al., 2022). Here, we wish to focus on the persistence of cultured T lymphocytes and their circulation in the blood, their homing to tissues and target cells, and their *in vivo* amplification after re-transfusion. All approved T cell therapies include the intravenous application of the T cell suspension. So far, limited data are available with regard to the circulation behavior of *ex vivo* expanded T cells including CAR T cells in the blood, their passage through the lungs, and the dynamics and the rate-limiting steps on their way to migrate into tissues.

To move out of the bloodstream and migrate into tissues, most normal leucocytes undergo several steps including margination, interaction with vessel wall cells (tethering), arrest at the vessel walls, polarization, and transendothelial migration to finally enter the interstitial compartment of target tissues (Nourshargh and Alon, 2014; Kameritsch and Renkawitz, 2020). Major classes of receptors involved in the interaction of T cells with vessel wall cells are selectins and selectin ligands, chemokine receptors, and integrins. Typically, tethering of T lymphocytes to the vessel wall is mediated by P- and/or E-selectin ligands on T lymphocytes. A major molecule on T cells conferring binding to endothelial expressed P and E selectin is P selectin glycoprotein ligand (PSGL)-1, which is glycosylated in mature T cells but not glycosylated in naive T cells (Ley and Kansas, 2004). Other moieties in T cells regulating selectin binding are CD43 or TIM1 (Fuhlbrigge et al., 2006; Angiari et al., 2014). It is currently not known whether culture-expanded T cells, and which subpopulation thereof, retain the ability to bind to P and E selectin in postcapillary venules or other vessels where extravasation may be induced. Also, the capability of expanded T cells including CAR T cells to bind through their integrin receptors under shear stress and the subsequent activation of rapid, chemokine-induced integrin activation and adhesion strengthening are currently incompletely understood.

A potential role for E-selectin ligands on CAR T cells has been described in an increase of cell-autonomous tetrasaccharide sialyl-Lewis X (sLeX)-decorated E-selectin ligands during culture-expansion of CAR T cells (Sánchez-Martínez et al., 2021). However, glycoengineered enforced sialofucosylation of E-selectin ligands which could restore E-selectin binding was found dispensable for CD19-CAR T-cell activity and BM homing in xenograft models. In contrast, Mondal et al. (2019), reported that external chemical fucosylation of expanded human CAR-T cells results in their infiltration of marrow with 10-fold higher efficiency than unfucosylated cells, leaving the question of the role of E-selectin in reaching the target tissue by CAR T cells open (Mondal et al., 2019).

It should be mentioned that the presence and composition of cytokines during the culture process influence development into different T cell subtypes, such as cytotoxic and central or effector memory cell types, which display different adhesion receptors. Gargett et al. (2019) demonstrated this when comparing an industrial CAR T cell expansion process with two improved culture protocols, in which the generation of CAR-T cells with enhanced CD62L and CCR7 expression was achieved (Gargett et al., 2019). A role for L-selectin in the function of retransfused CAR T cells has been investigated by Watson et al. (2019) by tracking Zirconium-89 (89Zr)–labeled T cells which expressed L-selectin or not (Watson et al., 2019). Using PET-CT, the authors showed that the transfused T cells localized to tumors between 1 hour and 7 days after transfusion. Although L-selectin did not promote T cell homing to tumors, the early activation marker CD69 was upregulated in the T cell expressing L-selectin but not in the L-selectin knockout T cells in their preclinical mouse model (Watson et al., 2019). Ngai et al. (2019) investigated the conditions governing the preservation of L-selectin (CD62L)–expressing T cells during *ex vivo* expansion of nonalloreactive tumor-reactive NKT cells (Ngai et al., 2018). IL-21 was found to protect the preservation of antitumor activity during the expansion process by downregulating the pro-apoptotic protein BIM in CAR T cells (Ngai et al., 2018). L-selectin expression was also found to increase in CAR T cells after inhibiting AKT signaling during culture, which was related to a higher proportion of more memory-type T cells (Klebanoff et al., 2017).

A further set of molecules which are intricately tied to T cell migration, including the activation of integrins to initiate the sudden arrest of cells flowing in the blood, are chemokine receptors (Alon and Feigelson, 2009). Chemokine receptors have been investigated in the context of CAR T cell function, yet mostly with a focus on their role in intra-tissue migration of the T cells, and will therefore be reviewed in the intra-tissue migration part below.

One of the early observations of CAR T cell toxicity pointing to activation of integrins has been a lethal complication early after CAR T cell infusion, with included capillary blockage by activated CAR T cells in conjunction with a so-called “cytokine storm” originating from the lungs (Morgan et al., 2010). The dynamics of the described reactions are highly suggestive of interactions between transfused cells and endothelial cells early after transfusion. Now widely accepted as a potentially severe side effect of CAR T cell therapy, cytokine release syndrome and its pathogenesis has been described earlier. A predictive role of the degree of endothelial cell activation in cytokine release syndrome has been found associated with levels of circulating soluble intercellular adhesion molecule (sICAM-1) and soluble vascular cell adhesion molecule (sVCAM)-1 (Hong et al., 2021). Thus, the interaction of endothelial integrin ligands with integrins may contribute to CAR T cell toxicity.

In conclusion, more investigations are needed to characterize the interaction patterns of expanded and CAR T cells with the vessel wall, for example in flow chamber *in vitro* models. *In vivo*, transfused CAR T cells have also been followed in the bloodstream. A steep increase in the transfused cell numbers has been observed, resembling extensive cell division after applications, which correlates with therapeutic success (Gauthier et al., 2020). It is not clear where the CAR T cells divide, however, most likely, outside the blood circulation.

### Intra-Tissue Migration

When moving the tissue compartment, T cells are among the softest and most flexible cell types known, able to relatively move fast within tissues with a velocity of 10–15 µm/min (Fowell and Kim, 2021). Upon activation, they polarize and coordinate and organize their movement through tight spaces through a number of physiological stimuli, including sensing of chemokines and coordination of the intracellular cytoskeletal machinery. This has been found to involve the activation of formin-like 1 protein and coordination of actin retrograde flow (Viola et al., 2008; Bufi et al., 2015; Yamada and Sixt, 2019; Stolp et al., 2020).

Signaling to the cytoskeleton may occur through Rho Family small GTPases and G protein–coupled receptor and other intracellular signaling pathways (Mastrogiovanni et al., 2020). Transduction of CARs and subsequent *ex vivo* expansion of T lymphocytes has been found to result in increased activation of phosphoinositol-3 kinase (PI3K), which could affect migration induction (Wallstabe et al., 2019). The development of an exhausted T cell phenotype, which has not only been observed during the culture process but also after transfusion in the host organism, has been observed and could be prevented in CAR T cells during the culture process by intermittent blockade of CAR signaling and transient rest and epigenetic remodeling by a signal transduction inhibitor dasatinib (Mamonkin and Brenner, 2021; Weber et al., 2021). Newick et al. (2016) described CAR T cells which express an inhibitor of the association of protein kinase A (PKA) with ezrin, resulting in improved TCR activation. This resulted in higher adhesion of the CAR T cells to various matrices and improved their trafficking into tumor sites (Newick et al., 2016). When the CXCR4 receptor, which has been described as crucial for the migration of various types of leukocytes, is overexpressed, the *in vitro* migration of CAR NK cells was found augmented (Jamali et al., 2020). Haran et al. (2018) engineered CXCR5 expressing CAR T cells and demonstrated their increased migration to the CXCR5 ligand CXCL13 and increased accumulation in B cell follicles *in vivo* (Haran et al., 2018). When the IL-8 receptor CXCR2 was expressed in CAR T cells, homing to IL-8 expressing tumors was induced in murine xenograft models of solid tumors and hepatocellular carcinoma (Whilding et al., 2019; Liu et al., 2020). A double genetic approach to support the migration of CAR T cells into solid tumors was introduced by Cadilha et al. (2020) who *de novo* expressed the chemokine receptor CCR8 plus a double negative Transforming Growth Factor (TGF)-beta, resulting in improved therapeutic efficiency (Cadilha et al., 2021). In a genetic screen, Rogers et al. (2020) identified a gene regulating chemokine receptor expression in T cells as one of the most potent factors influencing the efficacy of CAR T cells against tumors, indicating the crucial role of chemokine receptors and T cell migration in the functionality of CAR T cells (Rogers et al., 2020). Taken together, the current results suggest a role for chemokine receptors in the migration of CAR T cells toward target cells, and further investigation into the role of individual chemokine receptors in CAR T cells seems promising.

### Metabolism and Optimization of *ex vivo* Culture Conditions of the T cells Pre-Infusion

Physiologically, T cells generally use oxidative phosphorylation (OXPHOS) during rest and switch to glycolysis during expansion phases, that is, anabolic conditions (Jenkins et al., 2021). Since the cell culture process classically implies the usage of glucose-containing media, metabolic programs will be induced which simulate glycolysis during the T cell expansion. Glycolysis has been linked to potentially disadvantageous effects in the long term through the accumulation of lactate during the culture expansion process of therapeutically used T cells (Quinn et al., 2020). Attempts are, therefore, underway to render CAR T cells more flexible to adapt to competition for nutrients after application, for example, carbohydrates and amino acids within the tumor microenvironment and to counteract changes in mitochondrial function and development of T cell exhaustion which are induced by the tumor microenvironment (Jenkins et al., 2021).

CAR-T cells expressing a CD28 co-stimulatory domain (e.g. *icabtagene Ciloleucel*) were found to display enhanced aerobic glycolysis and effector memory differentiation, whereas the inclusion of a 4-1BB co-stimulatory domain instead of CD28 (in products *Tisagenlecleucel*, UCART19, *Lisocabtagene Maraleucel*) favors OXPHOS and mitochondrial function (Kawalekar et al., 2016).

Metabolic conditioning also includes the optimization of culture conditions toward differentiation of the starting T cell population into the most desired T cell phenotypes and functions. Possibilities include a shorter culture duration, glucose restriction, supplementation of carnosine and arginine, and increased expression of arginine resynthesizing enzymes (Kawalekar et al., 2016; Jenkins et al., 2021). Alterations in intracellular signaling pathways may be desirable such as the PI3K and PPARg pathways. An engineered reduction of glycolysis has been reached in TRUCKs (Petersen and Krenciute, 2019), enhanced OXPHOS, and enhanced respiratory capacity carnosine inclusion in media formulations, which favor OXPHOS and neutralize protons (Jenkins et al., 2021).

It has been shown that lactate impairs nicotinamide adenine dinucleotide (NAD+) regeneration and blocks T cell proliferation, which could be rescued through supplementation with serine to restore the redox balance (Quinn et al., 2020). Another possibility to counteract T cell inhibition has been to counteract the thriving of (suppressive) T regulatory cells during the culture process since regulatory T cells will induce immunosuppression and tumor immune evasion in the low-glucose, high-lactate tumor microenvironment or mitochondrial exhaustion in a low-oxygen environment (Jenkins et al., 2021).

Fewer data exist on the changes in intracellular metabolism during the initial expansion phase. Further strategies to improve CAR T cell function, differentiation, and/or persistence in culture have been recently reviewed by Jenkins et al. (2021) and include, among others, LDH inhibition, and inhibition of cholesterol esterification by the enzyme cholesterol acyltransferase 1 (ACAT1) utilizing the repurposed drug avasimibe (Yang et al., 2016). This may result in an increased number of T memory cells, arginine supplementation or expression of arginine resynthesizing enzymes, and glucose restriction, for example, galactose addition can increase enhanced effector capabilities including IFNg and granzyme B production (Yang et al., 2016). Other possibilities include the addition of inosine or of inhibitors of glutamine usage. Thus, metabolism-targeted intracellular cellular engineering of T cells during culture expansion is an area of ongoing development and utilization in biotechnology

## Conclusion

Overall, this review provides an overview of current culture conditions and their influence on the final CAR T cell product. We also shed light on underexplored areas in the field, such as endothelial cell activation associated with CAR T cell application, migration capabilities, and metabolism of CAR T cells. The reviewed literature indicates that culture conditions may have a major impact on the final CAR T cell product obtained for infusion. However, there are very few studies understanding the underlying cell biology impacted by the culture conditions, resulting in a specific phenotype of expanded T cells, which may or may not have a therapeutic impact. This leaves multiple avenues for further biotechnological research, leading this promising cellular therapy into the future.

## Abbreviation

R/R, relapsed or refractory.

## References

[B1] Abou-El-Enein M. Elsallab M. Feldman S. A. Fesnak A. D. Heslop H. E. Marks P. (2021). Scalable Manufacturing of CAR T Cells for Cancer Immunotherapy. Blood Cancer Discov. 2 (5), 408–422. 10.1158/2643-3230.bcd-21-0084 34568831 PMC8462122

[B2] Abramowski-Mock U. Delhove J. M. Qasim W. (2017). Gene Modified T Cell Therapies for Hematological Malignancies. Hematology/Oncology Clin. N. Am. 31 (5), 913–926. 10.1016/j.hoc.2017.06.005 28895856

[B3] Abramson J. S. Palomba M. L. Gordon L. I. Lunning M. A. Wang M. Arnason J. (2020). Lisocabtagene Maraleucel for Patients with Relapsed or Refractory Large B-Cell Lymphomas (TRANSCEND NHL 001): a Multicentre Seamless Design Study. Lancet 396 (10254), 839–852. 10.1016/s0140-6736(20)31366-0 32888407

[B4] Aghajanian H. Rurik J. G. Epstein J. A. (2022). CAR-based Therapies: Opportunities for Immuno-Medicine beyond Cancer. Nat. Metab. 4 (2), 163–169. 10.1038/s42255-022-00537-5 35228742 PMC9947862

[B5] Albert S. Arndt C. Feldmann A. Bergmann R. Bachmann D. Koristka S. (2017). A Novel Nanobody-Based Target Module for Retargeting of T Lymphocytes to EGFR-Expressing Cancer Cells via the Modular UniCAR Platform. Oncoimmunology 6 (4), e1287246. 10.1080/2162402x.2017.1287246 28507794 PMC5414885

[B6] Alnabhan R. Gaballa A. Mörk L.-M. Mattsson J. Uhlin M. Magalhaes I. (2018). Media Evaluation for Production and Expansion of Anti-CD19 Chimeric Antigen Receptor T Cells. Cytotherapy 20 (7), 941–951. 10.1016/j.jcyt.2018.04.007 29859774

[B7] Alon R. Feigelson S. W. (2009). Chemokine Signaling to Lymphocyte Integrins under Shear Flow. Microcirculation 16 (1), 3–16. 10.1080/10739680802026076 18608990

[B8] Angiari S. Donnarumma T. Rossi B. Dusi S. Pietronigro E. Zenaro E. (2014). TIM-1 Glycoprotein Binds the Adhesion Receptor P-Selectin and Mediates T Cell Trafficking during Inflammation and Autoimmunity. Immunity 40 (4), 542–553. 10.1016/j.immuni.2014.03.004 24703780 PMC4066214

[B9] Apel M. Brüning M. Granzin M. Essl M. Stuth J. Blaschke J. (2013). Integrated Clinical Scale Manufacturing System for Cellular Products Derived by Magnetic Cell Separation, Centrifugation and Cell Culture. Chem. Ing. Tech. 85 (1-2), 103–110. 10.1002/cite.201200175

[B10] Arndt C. Loureiro L. R. Feldmann A. Jureczek J. Bergmann R. Máthé D. (2020). UniCAR T Cell Immunotherapy Enables Efficient Elimination of Radioresistant Cancer Cells. Oncoimmunology 9 (1), 1743036. 10.1080/2162402x.2020.1743036 32426176 PMC7219270

[B11] Asao H. Interleukin-2☆. In: Reference Module in Biomedical Sciences. Elsevier; 4, 2014.

[B12] Bachmann D. Aliperta R. Bergmann R. Feldmann A. Koristka S. Arndt C. (2018). Retargeting of UniCAR T Cells with an *In Vivo* Synthesized Target Module Directed against CD19 Positive Tumor Cells. Oncotarget 9 (7), 7487–7500. 10.18632/oncotarget.23556 29484126 PMC5800918

[B13] Bachmann M. (2019). The UniCAR System: A Modular CAR T Cell Approach to Improve the Safety of CAR T Cells. Immunol. Lett. 211, 13–22. 10.1016/j.imlet.2019.05.003 31091431

[B14] Bajgain P. Mucharla R. Wilson J. Welch D. Anurathapan U. Liang B. (2014). Optimizing the Production of Suspension Cells Using the G-Rex "M" Series. Mol. Ther. - Methods & Clin. Dev. 1, 14015. 10.1038/mtm.2014.15 26015959 PMC4362380

[B15] Bajgain P. Tawinwung S. D’Elia L. Sukumaran S. Watanabe N. Hoyos V. (2018). CAR T Cell Therapy for Breast Cancer: Harnessing the Tumor Milieu to Drive T Cell Activation. J. Immunother. cancer 6 (1), 34. 10.1186/s40425-018-0347-5 29747685 PMC5944113

[B16] Barrett D. M. Grupp S. A. June C. H. (2015). Chimeric Antigen Receptor- and TCR-Modified T Cells Enter Main Street and Wall Street. J. I. 195 (3), 755–761. 10.4049/jimmunol.1500751 PMC4507286 26188068

[B17] Baudequin T. Nyland R. Ye H. (2021). Objectives, Benefits and Challenges of Bioreactor Systems for the Clinical-Scale Expansion of T Lymphocyte Cells. Biotechnol. Adv. 49, 107735. 10.1016/j.biotechadv.2021.107735 33781889

[B18] Berdeja J. G. Madduri D. Usmani S. Z. Jakubowiak A. Agha M. Cohen A. D. (2021). Ciltacabtagene Autoleucel, a B-Cell Maturation Antigen-Directed Chimeric Antigen Receptor T-Cell Therapy in Patients with Relapsed or Refractory Multiple Myeloma (CARTITUDE-1): a Phase 1b/2 Open-Label Study. Lancet 398 (10297), 314–324. 34175021 10.1016/S0140-6736(21)00933-8

[B19] Bere A. Denny L. Hanekom W. Burgers W. A. Passmore J. A. (2010). Comparison of Polyclonal Expansion Methods to Improve the Recovery of Cervical Cytobrush-Derived T Cells from the Female Genital Tract of HIV-Infected Women. J. Immunol. Methods 354 (1-2), 68–79. 10.1016/j.jim.2010.02.002 20149794 PMC2854893

[B20] Berglund S. Gertow J. Magalhaes I. Mattsson J. Uhlin M. (2013). Cord Blood T Cells Cultured with IL-7 in Addition to IL-2 Exhibit a Higher Degree of Polyfunctionality and Superior Proliferation Potential. J. Immunother. 36 (8), 432–441. 10.1097/cji.0b013e3182a802f6 23994891

[B21] Besser M. J. Schallmach E. Oved K. Treves A. J. Markel G. Reiter Y. (2009). Modifying Interleukin-2 Concentrations during Culture Improves Function of T Cells for Adoptive Immunotherapy. Cytotherapy 11 (2), 206–217. 10.1080/14653240802590391 19148842

[B22] Bms (2021). Cell Therapy Resources - Bristol Myers Squibb. Available at: https://www.bms.com/media/media-library/scientific-media-resources/cell-therapy.html (Accessed on April 12, 2022).

[B23] Bouchkouj N. Zimmerman M. Kasamon Y. L. Wang C. Dai T. Xu Z. (2022). FDA Approval Summary: Axicabtagene Ciloleucel for Relapsed or Refractory Follicular Lymphoma. Oncologist. 10.1093/oncolo/oyac054 PMC9255972 35403693

[B24] Bouchkouj N. Kasamon Y. L. de Claro R. A. George B. Lin X. Lee S. (2019). FDA Approval Summary: Axicabtagene Ciloleucel for Relapsed or Refractory Large B-Cell Lymphoma. Clin. Cancer Res. 25 (6), 1702–1708. 10.1158/1078-0432.ccr-18-2743 30413526

[B25] Brentjens R. J. Davila M. L. Riviere I. Park J. Wang X. Cowell L. G. (2013). CD19-targeted T Cells Rapidly Induce Molecular Remissions in Adults with Chemotherapy-Refractory Acute Lymphoblastic Leukemia. Sci. Transl. Med. 5 (177), 177ra38. 10.1126/scitranslmed.3005930 PMC3742551 23515080

[B26] Brindley D. A. Davie N. L. Culme-Seymour E. J. Mason C. Smith D. W. Rowley J. A. (2012). Peak Serum: Implications of Serum Supply for Cell Therapy Manufacturing. Regen. Med. 7 (1), 7–13. 10.2217/rme.11.112 22168489

[B27] Brudno J. N. Maric I. Hartman S. D. Rose J. J. Wang M. Lam N. (2018). T Cells Genetically Modified to Express an Anti-B-cell Maturation Antigen Chimeric Antigen Receptor Cause Remissions of Poor-Prognosis Relapsed Multiple Myeloma. Jco 36 (22), 2267–2280. 10.1200/jco.2018.77.8084 PMC6067798 29812997

[B28] Buechner J. Kersten M. J. Fuchs M. Salmon F. Jäger U. (2018). Chimeric Antigen Receptor-T Cell Therapy. Hemasphere 2 (1), e18. 10.1097/hs9.0000000000000018 31723747 PMC6745952

[B29] Bufi N. Saitakis M. Dogniaux S. Buschinger O. Bohineust A. Richert A. (2015). Human Primary Immune Cells Exhibit Distinct Mechanical Properties that Are Modified by Inflammation. Biophysical J. 108 (9), 2181–2190. 10.1016/j.bpj.2015.03.047 PMC4423053 25954876

[B30] Cadilha B. L. Benmebarek M. R. Dorman K. Oner A. Lorenzini T. Obeck H. (2021). Combined Tumor-Directed Recruitment and Protection from Immune Suppression Enable CAR T Cell Efficacy in Solid Tumors. Sci. Adv. 7 (24). 10.1126/sciadv.abi5781 PMC8189699 34108220

[B31] Canestrari E. Steidinger H. R. McSwain B. Charlebois S. J. Dann C. T. (2019). Human Platelet Lysate Media Supplement Supports Lentiviral Transduction and Expansion of Human T Lymphocytes while Maintaining Memory Phenotype. J. Immunol. Res. 2019, 3616120. 10.1155/2019/3616120 31565660 PMC6746159

[B32] Cartellieri M. Feldmann A. Koristka S. Arndt C. Loff S. Ehninger A. (2016). Switching CAR T Cells on and off: a Novel Modular Platform for Retargeting of T Cells to AML Blasts. Blood Cancer J. 6 (8), e458. 10.1038/bcj.2016.61 27518241 PMC5022178

[B33] Cartellieri M. Loff S. von Bonin M. Bejestani E. P. Ehninger A. Feldmann A. (2015). Unicar: A Novel Modular Retargeting Platform Technology for CAR T Cells. Blood 126 (23), 5549. 10.1182/blood.v126.23.5549.5549 PMC5022178 27518241

[B34] Caruana I. Savoldo B. Hoyos V. Weber G. Liu H. Kim E. S. (2015). Heparanase Promotes Tumor Infiltration and Antitumor Activity of CAR-Redirected T Lymphocytes. Nat. Med. 21 (5), 524–529. 10.1038/nm.3833 25849134 PMC4425589

[B35] Cha E. Graham L. Manjili M. H. Bear H. D. (2010). IL-7 + IL-15 Are Superior to IL-2 for the *Ex Vivo* Expansion of 4T1 Mammary Carcinoma-specific T Cells with Greater Efficacy against Tumors *In Vivo* . Breast Cancer Res. Treat. 122 (2), 359–369. 10.1007/s10549-009-0573-0 19826947 PMC4033304

[B36] Chicaybam L. Sodre A. L. Curzio B. A. Bonamino M. H. (2013). An Efficient Low Cost Method for Gene Transfer to T Lymphocytes. PLoS One 8 (3), e60298. 10.1371/journal.pone.0060298 23555950 PMC3608570

[B37] Chmielewski M. Abken H. (2020). TRUCKS, the Fourth‐generation CAR T Cells: Current Developments and Clinical Translation. Adv. Cell. Gene Ther. 3 (3). 10.1002/acg2.84

[B38] Chmielewski M. Abken H. (2015). TRUCKs: the Fourth Generation of CARs. Expert Opin. Biol. Ther. 15 (8), 1145–1154. 10.1517/14712598.2015.1046430 25985798

[B39] Clinicaltrials (2022). Search of: CAR T Cell - List Results - ClinicalTrials.Gov. Available from: https://www.clinicaltrials.gov/ct2/results?cond=CAR+T+cell&term=&cntry=&state=&city=&dist= .

[B40] Coeshott C. Vang B. Jones M. Nankervis B. (2019). Large-scale Expansion and Characterization of CD3+ T-Cells in the Quantum Cell Expansion System. J. Transl. Med. 17 (1), 258. 10.1186/s12967-019-2001-5 31391068 PMC6686483

[B41] Cosenza M. Sacchi S. Pozzi S. (2021). Cytokine Release Syndrome Associated with T-Cell-Based Therapies for Hematological Malignancies: Pathophysiology, Clinical Presentation, and Treatment. Int. J. Mol. Sci. 22 (14). 10.3390/ijms22147652 PMC8305850 34299273

[B42] Dafni U. Michielin O. Lluesma S. M. Tsourti Z. Polydoropoulou V. Karlis D. (2019). Efficacy of Adoptive Therapy with Tumor-Infiltrating Lymphocytes and Recombinant Interleukin-2 in Advanced Cutaneous Melanoma: a Systematic Review and Meta-Analysis. Ann. Oncol. 30 (12), 1902–1913. 10.1093/annonc/mdz398 31566658

[B43] Dasyam N. George P. Weinkove R. (2020). Chimeric Antigen Receptor T‐cell Therapies: Optimising the Dose. Br. J. Clin. Pharmacol. 86 (9), 1678–1689. 10.1111/bcp.14281 32175617 PMC7444796

[B44] Depil S. Duchateau P. Grupp S. A. Mufti G. Poirot L. (2020). 'Off-the-shelf' Allogeneic CAR T Cells: Development and Challenges. Nat. Rev. Drug Discov. 19 (3), 185–199. 10.1038/s41573-019-0051-2 31900462

[B45] Deshpande A. Rule W. Rosenthal A. (2022). Radiation and Chimeric Antigen Receptor T-Cell Therapy in B-Cell Non-hodgkin Lymphomas. Curr. Treat. Options Oncol. 23 (1), 89–98. 10.1007/s11864-021-00935-z 35167008

[B46] Dragon A. C. Zimmermann K. Nerreter T. Sandfort D. Lahrberg J. Klöß S. (2020). CAR-T Cells and TRUCKs that Recognize an EBNA-3c-Derived Epitope Presented on HLA-B*35 Control Epstein-Barr Virus-Associated Lymphoproliferation. J. Immunother. Cancer 8 (2), e000736. 10.1136/jitc-2020-000736 33127653 PMC7604878

[B47] Du S.-H. Li Z. Chen C. Tan W.-K. Chi Z. Kwang T. W. (2016). Co-Expansion of Cytokine-Induced Killer Cells and Vγ9Vδ2 T Cells for CAR T-Cell Therapy. PLoS One 11 (9), e0161820. 10.1371/journal.pone.0161820 27598655 PMC5012695

[B48] Dufva O. Koski J. Maliniemi P. Ianevski A. Klievink J. Leitner J. (2020). Integrated Drug Profiling and CRISPR Screening Identify Essential Pathways for CAR T-Cell Cytotoxicity. Blood 135 (9), 597–609. 10.1182/blood.2019002121 31830245 PMC7098811

[B49] Elavia N. Panch S. R. McManus A. Bikkani T. Szymanski J. Highfill S. L. (2019). Effects of Starting Cellular Material Composition on Chimeric Antigen Receptor T‐cell Expansion and Characteristics. Transfusion 59 (5), 1755–1764. 10.1111/trf.15287 30973976

[B50] Ellebrecht C. T. Bhoj V. G. Nace A. Choi E. J. Mao X. Cho M. J. (2016). Reengineering Chimeric Antigen Receptor T Cells for Targeted Therapy of Autoimmune Disease. Science 353 (6295), 179–184. 10.1126/science.aaf6756 27365313 PMC5343513

[B51] Ellebrecht C. T. Lundgren D. K. Payne A. S. (2019). On the Mark: Genetically Engineered Immunotherapies for Autoimmunity. Curr. Opin. Immunol. 61, 69–73. 10.1016/j.coi.2019.08.005 31563849 PMC6901707

[B52] Eyquem J. Mansilla-Soto J. Giavridis T. van der Stegen S. J. C. Hamieh M. Cunanan K. M. (2017). Targeting a CAR to the TRAC Locus with CRISPR/Cas9 Enhances Tumour Rejection. Nature 543 (7643), 113–117. 10.1038/nature21405 28225754 PMC5558614

[B53] Feau S. Arens R. Togher S. Schoenberger S. P. (2011). Autocrine IL-2 Is Required for Secondary Population Expansion of CD8+ Memory T Cells. Nat. Immunol. 12 (9), 908–913. 10.1038/ni.2079 21804558 PMC3388550

[B54] Feins S. Kong W. Williams E. F. Milone M. C. Fraietta J. A. (2019). An Introduction to Chimeric Antigen Receptor (CAR) T‐cell Immunotherapy for Human Cancer. Am. J. Hematol. 94 (S1), S3–S9. 10.1002/ajh.25418 30680780

[B55] Feldmann A. Arndt C. Bergmann R. Loff S. Cartellieri M. Bachmann D. (2017). Retargeting of T Lymphocytes to PSCA- or PSMA Positive Prostate Cancer Cells Using the Novel Modular Chimeric Antigen Receptor Platform Technology "UniCAR". Oncotarget 8 (19), 31368–31385. 10.18632/oncotarget.15572 28404896 PMC5458214

[B56] Festen R. (2007). “Understanding Animal Sera: Considerations for Use in the Production of Biological Therapeutics,” in Medicines from Animal Cell Culture. Editors Stacey G. Davis J. (Chichester, UK: John Wiley & Sons), 45–58.

[B57] Feuchtinger T. Lücke J. Hamprecht K. Richard C. Handgretinger R. Schumm M. (2005). Detection of Adenovirus-specific T Cells in Children with Adenovirus Infection after Allogeneic Stem Cell Transplantation. Br. J. Haematol. 128 (4), 503–509. 10.1111/j.1365-2141.2004.05331.x 15686459

[B58] Feuchtinger T. Matthes-Martin S. Richard C. Lion T. Fuhrer M. Hamprecht K. (2006). Safe Adoptive Transfer of Virus-specific T-Cell Immunity for the Treatment of Systemic Adenovirus Infection after Allogeneic Stem Cell Transplantation. Br. J. Haematol. 134 (1), 64–76. 10.1111/j.1365-2141.2006.06108.x 16803570

[B59] Fowell D. J. Kim M. (2021). The Spatio-Temporal Control of Effector T Cell Migration. Nat. Rev. Immunol. 21 (9), 582–596. 10.1038/s41577-021-00507-0 33627851 PMC9380693

[B60] Frey N. Porter D. (2019). Cytokine Release Syndrome with Chimeric Antigen Receptor T Cell Therapy. Biol. Blood Marrow Transplant. 25 (4), e123–e127. 10.1016/j.bbmt.2018.12.756 30586620

[B61] Fritsche E. Volk H.-D. Reinke P. Abou-El-Enein M. (2020). Toward an Optimized Process for Clinical Manufacturing of CAR-Treg Cell Therapy. Trends Biotechnol. 38 (10), 1099–1112. 10.1016/j.tibtech.2019.12.009 31982150

[B62] Fry T. J. Shah N. N. Orentas R. J. Stetler-Stevenson M. Yuan C. M. Ramakrishna S. (2018). CD22-targeted CAR T Cells Induce Remission in B-ALL that Is Naive or Resistant to CD19-Targeted CAR Immunotherapy. Nat. Med. 24 (1), 20–28. 10.1038/nm.4441 29155426 PMC5774642

[B63] Fuhlbrigge R. C. King S. L. Sackstein R. Kupper T. S. (2006). CD43 Is a Ligand for E-Selectin on CLA+ Human T Cells. Blood 107 (4), 1421–1426. 10.1182/blood-2005-05-2112 16269612 PMC1895405

[B64] Galetto R. Lebuhotel C. Poirot L. Gouble A. Toribio M. L. Smith J. (2014). Pre-Tcrα Supports CD3-dependent Reactivation and Expansion of TCRα-Deficient Primary Human T-Cells. Mol. Ther. - Methods & Clin. Dev. 1, 14021. 10.1038/mtm.2014.21 26015965 PMC4362381

[B65] Garcia Borrega J. Gödel P. Rüger M. A. Onur Ö. A. Shimabukuro-Vornhagen A. Kochanek M. (2019). In the Eye of the Storm: Immune-Mediated Toxicities Associated with CAR-T Cell Therapy. Hemasphere 3 (2), e191. 10.1097/hs9.0000000000000191 31723828 PMC6746039

[B66] Garcia-Aponte O. F. Herwig C. Kozma B. (2021). Lymphocyte Expansion in Bioreactors: Upgrading Adoptive Cell Therapy. J. Biol. Eng. 15 (1), 13. 10.1186/s13036-021-00264-7 33849630 PMC8042697

[B67] Gargett T. Truong N. Ebert L. M. Yu W. Brown M. P. (2019). Optimization of Manufacturing Conditions for Chimeric Antigen Receptor T Cells to Favor Cells with a Central Memory Phenotype. Cytotherapy 21 (6), 593–602. 10.1016/j.jcyt.2019.03.003 30975603

[B68] Gattinoni L. Lugli E. Ji Y. Pos Z. Paulos C. M. Quigley M. F. (2011). A Human Memory T Cell Subset with Stem Cell-like Properties. Nat. Med. 17 (10), 1290–1297. 10.1038/nm.2446 21926977 PMC3192229

[B69] Gauthier J. Hirayama A. V. Purushe J. Hay K. A. Lymp J. Li D. H. (2020). Feasibility and Efficacy of CD19-Targeted CAR T Cells with Concurrent Ibrutinib for CLL after Ibrutinib Failure. Blood 135 (19), 1650–1660. 10.1182/blood.2019002936 32076701 PMC7205814

[B70] Gauthier J. Turtle C. J. (2018). Insights into Cytokine Release Syndrome and Neurotoxicity after CD19-specific CAR-T Cell Therapy. Curr. Res. Transl. Med. 66 (2), 50–52. 10.1016/j.retram.2018.03.003 29625831 PMC5967886

[B71] Gavriilaki E. Sakellari I. Gavriilaki M. Anagnostopoulos A. (2020). A New Era in Endothelial Injury Syndromes: Toxicity of CAR-T Cells and the Role of Immunity. Int. J. Mol. Sci. 21 (11). 10.3390/ijms21113886 PMC7312228 32485958

[B72] Gee A. P. (2018). GMP CAR-T Cell Production. Best Pract. Res. Clin. Haematol. 31 (2), 126–134. 10.1016/j.beha.2018.01.002 29909913

[B73] Ghaffari S. Torabi-Rahvar M. Aghayan S. Jabbarpour Z. Moradzadeh K. Omidkhoda A. (2021). Optimizing Interleukin-2 Concentration, Seeding Density and Bead-To-Cell Ratio of T-Cell Expansion for Adoptive Immunotherapy. BMC Immunol. 22 (1), 43. 10.1186/s12865-021-00435-7 34217218 PMC8254233

[B74] Ghassemi S. Durgin J. S. Nunez-Cruz S. Patel J. Leferovich J. Pinzone M. (2022). Rapid Manufacturing of Non-activated Potent CAR T Cells. Nat. Biomed. Eng. 6 (2), 118–128. 10.1038/s41551-021-00842-6 35190680 PMC8860360

[B75] Ghassemi S. Martinez-Becerra F. J. Master A. M. Richman S. A. Heo D. Leferovich J. (2020). Enhancing Chimeric Antigen Receptor T Cell Anti-tumor Function through Advanced Media Design. Mol. Ther. - Methods & Clin. Dev. 18, 595–606. 10.1016/j.omtm.2020.07.008 32775494 PMC7397397

[B76] Golubovskaya V. Wu L. (2016). Different Subsets of T Cells, Memory, Effector Functions, and CAR-T Immunotherapy. Cancers (Basel) 8 (3). 10.3390/cancers8030036 PMC4810120 26999211

[B77] Grigor E. J. M. Fergusson D. Kekre N. Montroy J. Atkins H. Seftel M. D. (2019). Risks and Benefits of Chimeric Antigen Receptor T-Cell (CAR-T) Therapy in Cancer: A Systematic Review and Meta-Analysis. Transfus. Med. Rev. 33 (2), 98–110. 10.1016/j.tmrv.2019.01.005 30948292

[B78] Gross G. Waks T. Eshhar Z. (1989). Expression of Immunoglobulin-T-Cell Receptor Chimeric Molecules as Functional Receptors with Antibody-type Specificity. Proc. Natl. Acad. Sci. U.S.A. 86 (24), 10024–10028. 10.1073/pnas.86.24.10024 2513569 PMC298636

[B79] Grupp S. A. Kalos M. Barrett D. Aplenc R. Porter D. L. Rheingold S. R. (2013). Chimeric Antigen Receptor-Modified T Cells for Acute Lymphoid Leukemia. N. Engl. J. Med. 368 (16), 1509–1518. 10.1056/nejmoa1215134 23527958 PMC4058440

[B80] Gust J. Hay K. A. Hanafi L.-A. Li D. Myerson D. Gonzalez-Cuyar L. F. (2017). Endothelial Activation and Blood-Brain Barrier Disruption in Neurotoxicity after Adoptive Immunotherapy with CD19 CAR-T Cells. Cancer Discov. 7 (12), 1404–1419. 10.1158/2159-8290.cd-17-0698 29025771 PMC5718945

[B81] Haran K. P. Hajduczki A. Pampusch M. S. Mwakalundwa G. Vargas-Inchaustegui D. A. Rakasz E. G. (2018). Simian Immunodeficiency Virus (SIV)-Specific Chimeric Antigen Receptor-T Cells Engineered to Target B Cell Follicles and Suppress SIV Replication. Front. Immunol. 9, 492. 10.3389/fimmu.2018.00492 29616024 PMC5869724

[B82] Henschler R. (2018). “Cell Culture Media,” in Cell Culture Technology. Editors Kasper C. Charwat V. Lavrentieva A. (Cham, Switzerland: Springer), 49–59. 10.1007/978-3-319-74854-2_3

[B83] Holzinger A. Abken H. (2019). CAR T Cells: A Snapshot on the Growing Options to Design a CAR. Hemasphere 3 (1), e172. 10.1097/hs9.0000000000000172 31723811 PMC6745938

[B84] Hong F. Shi M. Cao J. Wang Y. Gong Y. Gao H. (2021). Predictive Role of Endothelial Cell Activation in Cytokine Release Syndrome after Chimeric Antigen Receptor T Cell Therapy for Acute Lymphoblastic Leukaemia. J. Cell. Mol. Med. 25 (24), 11063–11074. 10.1111/jcmm.17029 34734474 PMC8650023

[B85] Huang J. Kerstann K. W. Ahmadzadeh M. Li Y. F. El-Gamil M. Rosenberg S. A. (2006). Modulation by IL-2 of CD70 and CD27 Expression on CD8+ T Cells: Importance for the Therapeutic Effectiveness of Cell Transfer Immunotherapy. J. Immunol. 176 (12), 7726–7735. 10.4049/jimmunol.176.12.7726 16751420 PMC1532931

[B86] Huang R. Li X. He Y. Zhu W. Gao L. Liu Y. (2020). Recent Advances in CAR-T Cell Engineering. J. Hematol. Oncol. 13 (1), 86. 10.1186/s13045-020-00910-5 32616000 PMC7333410

[B87] Ikeda H. (2016). T-cell Adoptive Immunotherapy Using Tumor-Infiltrating T Cells and Genetically Engineered TCR-T Cells: Table 1. Intimm 28 (7), 349–353. 10.1093/intimm/dxw022 27127191

[B88] Jamali A. Hadjati J. Madjd Z. Mirzaei H. R. Thalheimer F. B. Agarwal S. (2020). Highly Efficient Generation of Transgenically Augmented CAR NK Cells Overexpressing CXCR4. Front. Immunol. 11, 2028. 10.3389/fimmu.2020.02028 32983147 PMC7483584

[B89] Janas M. Nunes C. Marenghi A. Sauvage V. (2015). Perfusion's Role in Maintenance of High-Density T-Cell Cultures. BioProcess Int. 13.

[B90] Jayaraman J. Mellody M. P. Hou A. J. Desai R. P. Fung A. W. Pham A. H. T. (2020). CAR-T Design: Elements and Their Synergistic Function. EBioMedicine 58, 102931. 10.1016/j.ebiom.2020.102931 32739874 PMC7393540

[B91] Jenkins Y. Zabkiewicz J. Ottmann O. Jones N. (2021). Tinkering under the Hood: Metabolic Optimisation of CAR-T Cell Therapy. Antibodies (Basel) 10 (2). 10.3390/antib10020017 PMC8167549 33925949

[B92] Johnson L. A. Scholler J. Ohkuri T. Kosaka A. Patel P. R. McGettigan S. E. (2015). Rational Development and Characterization of Humanized Anti-EGFR Variant III Chimeric Antigen Receptor T Cells for Glioblastoma. Sci. Transl. Med. 7 (275), 275ra22. 10.1126/scitranslmed.aaa4963 PMC4467166 25696001

[B93] Jureczek J. Bergmann R. Berndt N. Koristka S. Kegler A. Puentes-Cala E. (2019). An Oligo-His-Tag of a Targeting Module Does Not Influence its Biodistribution and the Retargeting Capabilities of UniCAR T Cells. Sci. Rep. 9 (1), 10547. 10.1038/s41598-019-47044-4 31332252 PMC6646371

[B94] Kalos M. Levine B. L. Porter D. L. Katz S. Grupp S. A. Bagg A. (2011). T Cells with Chimeric Antigen Receptors Have Potent Antitumor Effects and Can Establish Memory in Patients with Advanced Leukemia. Sci. Transl. Med. 3 (95), 95ra73. 10.1126/scitranslmed.3002842 PMC3393096 21832238

[B95] Kameritsch P. Renkawitz J. (2020). Principles of Leukocyte Migration Strategies. Trends Cell. Biol. 30 (10), 818–832. 10.1016/j.tcb.2020.06.007 32690238

[B96] Kang S. Tanaka T. Narazaki M. Kishimoto T. (2019). Targeting Interleukin-6 Signaling in Clinic. Immunity 50 (4), 1007–1023. 10.1016/j.immuni.2019.03.026 30995492

[B97] Kawalekar O. U. O’Connor R. S. Fraietta J. A. Guo L. McGettigan S. E. Posey A. D. (2016). Distinct Signaling of Coreceptors Regulates Specific Metabolism Pathways and Impacts Memory Development in CAR T Cells. Immunity 44 (2), 380–390. 10.1016/j.immuni.2016.01.021 26885860 PMC13498396

[B98] Kenderian S. S. Ruella M. Shestova O. Kim M. Klichinsky M. Chen F. (2017). Ruxolitinib Prevents Cytokine Release Syndrome after Car T-Cell Therapy without Impairing the Anti-tumor Effect in a Xenograft Model. Biol. Blood Marrow Transplant. 23 (3), S19–S20. 10.1016/j.bbmt.2016.12.003

[B99] Kim H. P. Imbert J. Leonard W. J. (2006). Both integrated and Differential Regulation of Components of the IL-2/IL-2 Receptor System. Cytokine & Growth Factor Rev. 17 (5), 349–366. 10.1016/j.cytogfr.2006.07.003 16911870

[B100] Kim M. S. Ma J. S. Y. Yun H. Cao Y. Kim J. Y. Chi V. (2015). Redirection of Genetically Engineered CAR-T Cells Using Bifunctional Small Molecules. J. Am. Chem. Soc. 137 (8), 2832–2835. 10.1021/jacs.5b00106 25692571

[B101] Kim N. Nam Y.-S. Im K.-I. Lim J.-Y. Jeon Y.-W. Song Y. (2018). Robust Production of Cytomegalovirus Pp65-specific T Cells Using a Fully Automated IFN-γ Cytokine Capture System. Transfus. Med. Hemother 45 (1), 13–22. 10.1159/000479238 29593456 PMC5836230

[B102] Klebanoff C. A. Crompton J. G. Leonardi A. J. Yamamoto T. N. Chandran S. S. Eil R. L. (2017). Inhibition of AKT Signaling Uncouples T Cell Differentiation from Expansion for Receptor-Engineered Adoptive Immunotherapy. JCI Insight 2 (23). 10.1172/jci.insight.95103 PMC5752304 29212954

[B103] Kravets V. G. Zhang Y. Sun H. (2017). Chimeric-Antigen-Receptor (CAR) T Cells and the Factors Influencing Their Therapeutic Efficacy. J. Immunol. Res. Ther. 2 (1), 100–113. 30443604 PMC6233887

[B104] Lee D. W. Gardner R. Porter D. L. Louis C. U. Ahmed N. Jensen M. (2014). Current Concepts in the Diagnosis and Management of Cytokine Release Syndrome. Blood 124 (2), 188–195. 10.1182/blood-2014-05-552729 24876563 PMC4093680

[B105] Lee D. W. Kochenderfer J. N. Stetler-Stevenson M. Cui Y. K. Delbrook C. Feldman S. A. (2015). T Cells Expressing CD19 Chimeric Antigen Receptors for Acute Lymphoblastic Leukaemia in Children and Young Adults: a Phase 1 Dose-Escalation Trial. Lancet 385 (9967), 517–528. 10.1016/s0140-6736(14)61403-3 25319501 PMC7065359

[B106] Lee D. W. Santomasso B. D. Locke F. L. Ghobadi A. Turtle C. J. Brudno J. N. (2019). ASTCT Consensus Grading for Cytokine Release Syndrome and Neurologic Toxicity Associated with Immune Effector Cells. Biol. Blood Marrow Transplant. 25 (4), 625–638. 10.1016/j.bbmt.2018.12.758 30592986 PMC12180426

[B107] Leick M. B. Maus M. V. Frigault M. J. (2021). Clinical Perspective: Treatment of Aggressive B Cell Lymphomas with FDA-Approved CAR-T Cell Therapies. Mol. Ther. 29 (2), 433–441. 10.1016/j.ymthe.2020.10.022 33130313 PMC7854294

[B108] Leney-Greene M. A. Boddapati A. K. Su H. C. Cantor J. R. Lenardo M. J. (2020). Human Plasma-like Medium Improves T Lymphocyte Activation. iScience 23 (1), 100759. 10.1016/j.isci.2019.100759 31887663 PMC6941860

[B109] Levine B. L. (2015). Performance-enhancing Drugs: Design and Production of Redirected Chimeric Antigen Receptor (CAR) T Cells. Cancer Gene Ther. 22 (2), 79–84. 10.1038/cgt.2015.5 25675873

[B110] Ley K. Kansas G. S. (2004). Selectins in T-Cell Recruitment to Non-lymphoid Tissues and Sites of Inflammation. Nat. Rev. Immunol. 4 (5), 325–336. 10.1038/nri1351 15122198

[B111] Li S. Wang X. Yuan Z. Liu L. Luo L. Li Y. (2021). Eradication of T-ALL Cells by CD7-Targeted Universal CAR-T Cells and Initial Test of Ruxolitinib-Based CRS Management. Clin. Cancer Res. 27 (5), 1242–1246. 10.1158/1078-0432.ccr-20-1271 33234511

[B112] Lin H. Cheng J. Mu W. Zhou J. Zhu L. (2021). Advances in Universal CAR-T Cell Therapy. Front. Immunol. 12, 744823. 10.3389/fimmu.2021.744823 34691052 PMC8526896

[B113] Liu G. Rui W. Zheng H. Huang D. Yu F. Zhang Y. (2020). CXCR2‐modified CAR‐T Cells Have Enhanced Trafficking Ability that Improves Treatment of Hepatocellular Carcinoma. Eur. J. Immunol. 50 (5), 712–724. 10.1002/eji.201948457 31981231

[B114] Lock D. Mockel-Tenbrinck N. Drechsel K. Barth C. Mauer D. Schaser T. (2017). Automated Manufacturing of Potent CD20-Directed Chimeric Antigen Receptor T Cells for Clinical Use. Hum. Gene Ther. 28 (10), 914–925. 10.1089/hum.2017.111 28847167

[B115] Long A. H. Haso W. M. Shern J. F. Wanhainen K. M. Murgai M. Ingaramo M. (2015). 4-1BB Costimulation Ameliorates T Cell Exhaustion Induced by Tonic Signaling of Chimeric Antigen Receptors. Nat. Med. 21 (6), 581–590. 10.1038/nm.3838 25939063 PMC4458184

[B116] Loureiro L. R. Feldmann A. Bergmann R. Koristka S. Berndt N. Arndt C. (2018). Development of a Novel Target Module Redirecting UniCAR T Cells to Sialyl Tn-Expressing Tumor Cells. Blood Cancer J. 8 (9), 81. 10.1038/s41408-018-0113-4 30190468 PMC6127150

[B117] Lu T. L. Pugach O. Somerville R. Rosenberg S. A. Kochenderfer J. N. Better M. (2016). A Rapid Cell Expansion Process for Production of Engineered Autologous CAR-T Cell Therapies. Hum. Gene Ther. Methods 27 (6), 209–218. 10.1089/hgtb.2016.120 27897048 PMC6445175

[B118] Ludwig J. Hirschel M. (2020). “Methods and Process Optimization for Large-Scale CAR T Expansion Using the G-Rex Cell Culture Platform,” in Chimeric Antigen Receptor T Cells: Development and Production/Edited by Kamilla Swiech, Kelen Cristina Ribeiro Malmegrim, Virgínia Picanço-Castro. Editors Swiech K. Ribeiro Malmegrim K. C. Picanco-Castro V. (New York: Humana Press), 165–177. 10.1007/978-1-0716-0146-4_12 31707675

[B119] Luostarinen A. Kaartinen T. Maliniemi P. Keto J. Arvas M. Belt H. (2017). Low IL-2 Concentration Favors Generation of Early Memory T Cells over Terminal Effectors during CAR T-Cell Expansion. Cytotherapy 19 (5), S8. 10.1016/j.jcyt.2017.02.008 28411126

[B120] Mahnke Y. D. Brodie T. M. Sallusto F. Roederer M. Lugli E. (2013). The Who's Who of T-Cell Differentiation: Human Memory T-Cell Subsets. Eur. J. Immunol. 43 (11), 2797–2809. 10.1002/eji.201343751 24258910

[B121] Mamonkin M. Brenner M. K. (2021). Reversal of Exhaustion in Engineered T Cells. Science 372 (6537), 34–35. 10.1126/science.abh0583 33795449

[B122] Marton C. Mercier-Letondal P. Galaine J. Godet Y. (2021). An Unmet Need: Harmonization of IL-7 and IL-15 Combination for the *Ex Vivo* Generation of Minimally Differentiated T Cells. Cell. Immunol. 363, 104314. 10.1016/j.cellimm.2021.104314 33677140

[B123] Mastrogiovanni M. Juzans M. Alcover A. Di Bartolo V. (2020). Coordinating Cytoskeleton and Molecular Traffic in T Cell Migration, Activation, and Effector Functions. Front. Cell. Dev. Biol. 8, 591348. 10.3389/fcell.2020.591348 33195256 PMC7609836

[B124] Medvec A. R. Ecker C. Kong H. Winters E. A. Glover J. Varela-Rohena A. (2018). Improved Expansion and *In Vivo* Function of Patient T Cells by a Serum-free Medium. Mol. Ther. - Methods & Clin. Dev. 8, 65–74. 10.1016/j.omtm.2017.11.001 29687031 PMC5907749

[B125] Meng H. Sun X. Song Y. Zou J. An G. Jin Z. (2018). La/SSB Chimeric Autoantibody Receptor Modified NK92MI Cells for Targeted Therapy of Autoimmune Disease. Clin. Immunol. 192, 40–49. 10.1016/j.clim.2018.04.006 29673902

[B126] Meng J. Wu X. Sun Z. Xun R. Liu M. Hu R. (2021). Efficacy and Safety of CAR-T Cell Products Axicabtagene Ciloleucel, Tisagenlecleucel, and Lisocabtagene Maraleucel for the Treatment of Hematologic Malignancies: A Systematic Review and Meta-Analysis. Front. Oncol. 11, 698607. 10.3389/fonc.2021.698607 34381720 PMC8350577

[B127] Met Ö. Jensen K. M. Chamberlain C. A. Donia M. Svane I. M. (2019). Principles of Adoptive T Cell Therapy in Cancer. Semin. Immunopathol. 41 (1), 49–58. 10.1007/s00281-018-0703-z 30187086

[B128] Mock U. Nickolay L. Philip B. Cheung G. W.-K. Zhan H. Johnston I. C. D. (2016). Automated Manufacturing of Chimeric Antigen Receptor T Cells for Adoptive Immunotherapy Using CliniMACS Prodigy. Cytotherapy 18 (8), 1002–1011. 10.1016/j.jcyt.2016.05.009 27378344

[B129] Mondal N. Silva M. Castano A. P. Maus M. V. Sackstein R. (2019). Glycoengineering of Chimeric Antigen Receptor (CAR) T-Cells to Enforce E-Selectin Binding. J. Biol. Chem. 294 (48), 18465–18474. 10.1074/jbc.ra119.011134 31628196 PMC6885642

[B130] Morgan R. A. Yang J. C. Kitano M. Dudley M. E. Laurencot C. M. Rosenberg S. A. (2010). Case Report of a Serious Adverse Event Following the Administration of T Cells Transduced with a Chimeric Antigen Receptor Recognizing ERBB2. Mol. Ther. 18 (4), 843–851. 10.1038/mt.2010.24 20179677 PMC2862534

[B131] Mueller Y. M. Makar V. Bojczuk P. M. Witek J. Katsikis P. D. (2003). IL-15 Enhances the Function and Inhibits CD95/Fas-Induced Apoptosis of Human CD4+ and CD8+ Effector-Memory T Cells. Int. Immunol. 15 (1), 49–58. 10.1093/intimm/dxg013 12502725

[B132] Murthy H. Iqbal M. Chavez J. C. Kharfan-Dabaja M. A. (2019). Cytokine Release Syndrome: Current Perspectives. Itt 8, 43–52. 10.2147/itt.s202015 31754614 PMC6825470

[B133] Neelapu S. S. Locke F. L. Bartlett N. L. Lekakis L. J. Miklos D. B. Jacobson C. A. (2017). Axicabtagene Ciloleucel CAR T-Cell Therapy in Refractory Large B-Cell Lymphoma. N. Engl. J. Med. 377 (26), 2531–2544. 10.1056/NEJMoa1707447 29226797 PMC5882485

[B134] Neelapu S. S. Tummala S. Kebriaei P. Wierda W. Gutierrez C. Locke F. L. (2018). Chimeric Antigen Receptor T-Cell Therapy - Assessment and Management of Toxicities. Nat. Rev. Clin. Oncol. 15 (1), 47–62. 10.1038/nrclinonc.2017.148 28925994 PMC6733403

[B135] Newick K. O'Brien S. Sun J. Kapoor V. Maceyko S. Lo A. (2016). Augmentation of CAR T-Cell Trafficking and Antitumor Efficacy by Blocking Protein Kinase A Localization. Cancer Immunol. Res. 4 (6), 541–551. 10.1158/2326-6066.cir-15-0263 27045023 PMC4891259

[B136] Ngai H. Tian G. Courtney A. N. Ravari S. B. Guo L. Liu B. (2018). IL-21 Selectively Protects CD62L+NKT Cells and Enhances Their Effector Functions for Adoptive Immunotherapy. J. I. 201 (7), 2141–2153. 10.4049/jimmunol.1800429 PMC6143411 30111631

[B137] Norelli M. Camisa B. Barbiera G. Falcone L. Purevdorj A. Genua M. (2018). Monocyte-derived IL-1 and IL-6 Are Differentially Required for Cytokine-Release Syndrome and Neurotoxicity Due to CAR T Cells. Nat. Med. 24 (6), 739–748. 10.1038/s41591-018-0036-4 29808007

[B138] Nourshargh S. Alon R. (2014). Leukocyte Migration into Inflamed Tissues. Immunity 41 (5), 694–707. 10.1016/j.immuni.2014.10.008 25517612

[B139] Novartis (2021). Novartis Expands Kymriah® Manufacturing Footprint with First-Ever Approved Site for Commercial CAR-T Cell Therapy Manufacturing in Asia. Available at: https://www.novartis.com/news/media-releases/novartis-expands-kymriah-manufacturing-footprint-first-ever-approved-site-commercial-car-t-cell-therapy-manufacturing-asia (Accessed on April 12, 2022).

[B140] O'Leary M. C. Lu X. Huang Y. Lin X. Mahmood I. Przepiorka D. (2019). FDA Approval Summary: Tisagenlecleucel for Treatment of Patients with Relapsed or Refractory B-Cell Precursor Acute Lymphoblastic Leukemia. Clin. Cancer Res. 25 (4), 1142–1146. 10.1158/1078-0432.ccr-18-2035 30309857

[B141] Ochi T. Fujiwara H. Yasukawa M. (2010). Application of Adoptive T-Cell Therapy Using Tumor Antigen-specific T-Cell Receptor Gene Transfer for the Treatment of Human Leukemia. J. Biomed. Biotechnol. 2010, 521248. 10.1155/2010/521248 20454585 PMC2864513

[B142] Page A. V. Liles W. C. (2013). Biomarkers of Endothelial Activation/dysfunction in Infectious Diseases. Virulence 4 (6), 507–516. 10.4161/viru.24530 23669075 PMC5359744

[B143] Pampusch M. S. Haran K. P. Hart G. T. Rakasz E. G. Rendahl A. K. Berger E. A. (2020). Rapid Transduction and Expansion of Transduced T Cells with Maintenance of Central Memory Populations. Mol. Ther. - Methods & Clin. Dev. 16, 1–10. 10.1016/j.omtm.2019.09.007 31673565 PMC6816036

[B144] Pan J. Deng B. Ling Z. Song W. Xu J. Duan J. (2021). Ruxolitinib Mitigates Steroid‐refractory CRS during CAR T Therapy. J. Cell. Mol. Med. 25 (2), 1089–1099. 10.1111/jcmm.16176 33314568 PMC7812291

[B145] Parameswaran N. Patial S. (2010). Tumor Necrosis Factor-α Signaling in Macrophages. Crit. Rev. Eukar Gene Expr. 20 (2), 87–103. 10.1615/critreveukargeneexpr.v20.i2.10 PMC3066460 21133840

[B146] Park J. H. Geyer M. B. Brentjens R. J. (2016). CD19-targeted CAR T-Cell Therapeutics for Hematologic Malignancies: Interpreting Clinical Outcomes to Date. Blood 127 (26), 3312–3320. 10.1182/blood-2016-02-629063 27207800 PMC4929923

[B147] Park J. H. Rivière I. Gonen M. Wang X. Sénéchal B. Curran K. J. (2018). Long-Term Follow-Up of CD19 CAR Therapy in Acute Lymphoblastic Leukemia. N. Engl. J. Med. 378 (5), 449–459. 10.1056/nejmoa1709919 29385376 PMC6637939

[B148] Petersen C. T. Krenciute G. (2019). Next Generation CAR T Cells for the Immunotherapy of High-Grade Glioma. Front. Oncol. 9, 69. 10.3389/fonc.2019.00069 30863720 PMC6399104

[B149] Pharma (2021). The Cocoon Platform Combined with the 4D-Nucleofector LV Unit. Available at: https://pharma.lonza.com/kc/CGT-KC-items/Cocoon-Platform-integrated-with%20the-4D-Nucleofector_PMD (Accessed on April 12, 2022).

[B150] Pharma.lonza (2022). The Cocoon® | Lonza. The Cocoon® | Lonza. Available at: https://pharma.lonza.com/technologies-products/cocoon-platform/cocoon (Accessed on April 12, 2022).

[B151] Pinnix C. C. Gunther J. R. Dabaja B. S. Strati P. Fang P. Hawkins M. C. (2020). Bridging Therapy Prior to Axicabtagene Ciloleucel for Relapsed/refractory Large B-Cell Lymphoma. Blood Adv. 4 (13), 2871–2883. 10.1182/bloodadvances.2020001837 32589728 PMC7362355

[B152] Pinz K. Liu H. Golightly M. Jares A. Lan F. Zieve G. W. (2016). Preclinical Targeting of Human T-Cell Malignancies Using CD4-specific Chimeric Antigen Receptor (CAR)-engineered T Cells. Leukemia 30 (3), 701–707. 10.1038/leu.2015.311 26526988

[B153] Pober J. S. Cotran R. S. (1990). The Role of Endothelial Cells in Inflammation. Transplantation 50 (4), 537–544. 10.1097/00007890-199010000-00001 2219269

[B154] Pober J. S. Sessa W. C. (2007). Evolving Functions of Endothelial Cells in Inflammation. Nat. Rev. Immunol. 7 (10), 803–815. 10.1038/nri2171 17893694

[B155] Porter D. Frey N. Wood P. A. Weng Y. Grupp S. A. (2018). Grading of Cytokine Release Syndrome Associated with the CAR T Cell Therapy Tisagenlecleucel. J. Hematol. Oncol. 11 (1), 35. 10.1186/s13045-018-0571-y 29499750 PMC5833070

[B156] Priesner C. Esser R. Tischer S. Marburger M. Aleksandrova K. Maecker-Kolhoff B. (2016). Comparative Analysis of Clinical-Scale IFN-γ-Positive T-Cell Enrichment Using Partially and Fully Integrated Platforms. Front. Immunol. 7, 393. 10.3389/fimmu.2016.00393 27746781 PMC5044705

[B157] Ptáčková P. Musil J. Štach M. Lesný P. Němečková Š. Král V. (2018). A New Approach to CAR T-Cell Gene Engineering and Cultivation Using piggyBac Transposon in the Presence of IL-4, IL-7 and IL-21. Cytotherapy 20 (4), 507–520. 29475789 10.1016/j.jcyt.2017.10.001

[B158] Quinn W. J. Jiao J. TeSlaa T. Stadanlick J. Wang Z. Wang L. (2020). Lactate Limits T Cell Proliferation via the NAD(H) Redox State. Cell. Rep. 33 (11), 108500. 10.1016/j.celrep.2020.108500 33326785 PMC7830708

[B159] Ramanathan S. Gagnon J. Dubois S. Forand-Boulerice M. Richter M. V. Ilangumaran S. (2009). Cytokine Synergy in Antigen-independent Activation and Priming of Naive CD8+ T Lymphocytes. Crit. Rev. Immunol. 29 (3), 219–239. 10.1615/critrevimmunol.v29.i3.30 19538136

[B160] Ramanathan S. Gagnon J. Ilangumaran S. (2008). Antigen-nonspecific Activation of CD8+ T Lymphocytes by Cytokines: Relevance to Immunity, Autoimmunity, and Cancer. Arch. Immunol. Ther. Exp. 56 (5), 311–323. 10.1007/s00005-008-0033-2 18836862

[B161] Rath J. A. Arber C. (2020). Engineering Strategies to Enhance TCR-Based Adoptive T Cell Therapy. Cells 9 (6). 10.3390/cells9061485 PMC7349724 32570906

[B162] Rauser G. Einsele H. Sinzger C. Wernet D. Kuntz G. Assenmacher M. (2004). Rapid Generation of Combined CMV-specific CD4+ and CD8+ T-Cell Lines for Adoptive Transfer into Recipients of Allogeneic Stem Cell Transplants. Blood 103 (9), 3565–3572. 10.1182/blood-2003-09-3056 14670917

[B163] Rogers L. M. Wang Z. Mott S. L. Dupuy A. J. Weiner G. J. (2020). A Genetic Screen to Identify Gain- and Loss-Of-Function Modifications that Enhance T-Cell Infiltration into Tumors. Cancer Immunol. Res. 8 (9), 1206–1214. 10.1158/2326-6066.cir-20-0056 32611665 PMC7483799

[B164] Roschewski M. Longo D. L. Wilson W. H. (2022). CAR T-Cell Therapy for Large B-Cell Lymphoma - Who, when, and How? N. Engl. J. Med. 386 (7), 692–696. 10.1056/nejme2118899 34904797 PMC9295142

[B165] Rose-John S. (2012). IL-6 Trans-signaling via the Soluble IL-6 Receptor: Importance for the Pro-inflammatory Activities of IL-6. Int. J. Biol. Sci. 8 (9), 1237–1247. 10.7150/ijbs.4989 23136552 PMC3491447

[B166] Rose-John S. Scheller J. Elson G. Jones S. A. (2006). Interleukin-6 Biology Is Coordinated by Membrane-Bound and Soluble Receptors: Role in Inflammation and Cancer. J. Leukoc. Biol. 80 (2), 227–236. 10.1189/jlb.1105674 16707558

[B167] Ross S. H. Cantrell D. A. (2018). Signaling and Function of Interleukin-2 in T Lymphocytes. Annu. Rev. Immunol. 36, 411–433. 10.1146/annurev-immunol-042617-053352 29677473 PMC6472684

[B168] Sadeqi Nezhad M. Seifalian A. Bagheri N. Yaghoubi S. Karimi M. H. Adbollahpour-Alitappeh M. (2020). Chimeric Antigen Receptor Based Therapy as a Potential Approach in Autoimmune Diseases: How Close Are We to the Treatment? Front. Immunol. 11, 603237. 10.3389/fimmu.2020.603237 33324420 PMC7727445

[B169] Sánchez-Martínez D. Gutiérrez-Agüera F. Romecin P. Vinyoles M. Palomo M. Tirado N. (2021). Enforced sialyl-Lewis-X (sLeX) Display in E-Selectin Ligands by Exofucosylation Is Dispensable for CD19-CAR T-Cell Activity and Bone Marrow Homing. Clin. Transl. Med. 11 (2), e280. 33634970 10.1002/ctm2.280 PMC7901721

[B170] Santomasso B. D. Park J. H. Salloum D. Riviere I. Flynn J. Mead E. (2018). Clinical and Biological Correlates of Neurotoxicity Associated with CAR T-Cell Therapy in Patients with B-Cell Acute Lymphoblastic Leukemia. Cancer Discov. 8 (8), 958–971. 10.1158/2159-8290.cd-17-1319 29880584 PMC6385599

[B171] Sato K. Kondo M. Sakuta K. Hosoi A. Noji S. Sugiura M. (2009). Impact of Culture Medium on the Expansion of T Cells for Immunotherapy. Cytotherapy 11 (7), 936–946. 10.3109/14653240903219114 19903105

[B172] Savoldo B. Ramos C. A. Liu E. Mims M. P. Keating M. J. Carrum G. (2011). CD28 Costimulation Improves Expansion and Persistence of Chimeric Antigen Receptor-Modified T Cells in Lymphoma Patients. J. Clin. Investig. 121 (5), 1822–1826. 10.1172/jci46110 21540550 PMC3083795

[B173] Schuster S. J. Maziarz R. T. Rusch E. S. Li J. Signorovitch J. E. Romanov V. V. (2020). Grading and Management of Cytokine Release Syndrome in Patients Treated with Tisagenlecleucel in the JULIET Trial. Blood Adv. 4 (7), 1432–1439. 10.1182/bloodadvances.2019001304 32271899 PMC7160283

[B174] Schuster S. J. Tam C. S. Borchmann P. Worel N. McGuirk J. P. Holte H. (2021). Long-term Clinical Outcomes of Tisagenlecleucel in Patients with Relapsed or Refractory Aggressive B-Cell Lymphomas (JULIET): a Multicentre, Open-Label, Single-Arm, Phase 2 Study. Lancet Oncol. 22 (10), 1403–1415. 10.1016/s1470-2045(21)00375-2 34516954

[B175] Seder R. A. Ahmed R. (2003). Similarities and Differences in CD4+ and CD8+ Effector and Memory T Cell Generation. Nat. Immunol. 4 (9), 835–842. 10.1038/ni969 12942084

[B176] Shah B. D. Ghobadi A. Oluwole O. O. Logan A. C. Boissel N. Cassaday R. D. (2021). KTE-X19 for Relapsed or Refractory Adult B-Cell Acute Lymphoblastic Leukaemia: Phase 2 Results of the Single-Arm, Open-Label, Multicentre ZUMA-3 Study. Lancet 398 (10299), 491–502. 10.1016/s0140-6736(21)01222-8 34097852 PMC11613962

[B177] Sharma P. Kanapuru B. George B. Lin X. Xu Z. Bryan W. W. (2022). FDA Approval Summary: Idecabtagene Vicleucel for Relapsed or Refractory Multiple Myeloma. Clin. Cancer Res. 10.1158/1078-0432.ccr-21-3803 PMC9064878 35046063

[B178] Shi Y. Wu W. Wan T. Liu Y. Peng G. Chen Z. (2013). Impact of Polyclonal Anti-cd3/cd28-coated Magnetic Bead Expansion Methods on T Cell Proliferation, Differentiation and Function. Int. Immunopharmacol. 15 (1), 129–137. 10.1016/j.intimp.2012.10.023 23159335

[B179] Shimabukuro-Vornhagen A. Gödel P. Subklewe M. Stemmler H. J. Schlößer H. A. Schlaak M. (2018). Cytokine Release Syndrome. J. Immunother. cancer 6 (1), 56. 10.1186/s40425-018-0343-9 29907163 PMC6003181

[B180] Shimabukuro-Vornhagen A. Gödel P. Subklewe M. Stemmler H. J. Schlößer H. A. Schlaak M. (2018). Cytokine Release Syndrome. J. Immunother. cancer 6 (1), 56. 10.1186/s40425-018-0343-9 29907163 PMC6003181

[B181] Si S. Teachey D. T. (2020). Spotlight on Tocilizumab in the Treatment of CAR-T-Cell-Induced Cytokine Release Syndrome: Clinical Evidence to Date. Ther. Clin. Risk Manag. 16, 705–714. 10.2147/TCRM.S223468 32801727 PMC7414980

[B182] SiddiqiT.SoumeraiJ. D.DorritieK. A.StephensD. M.RiedellP. A.ArnasonJ. (2022). Phase 1 TRANSCEND CLL 004 Study of Lisocabtagene Maraleucel in Patients with Relapsed/refractory CLL or SLL. Blood 139 (12), 1794–1806. 10.1182/blood.2021011895 34699592

[B183] Siegler E. L. Kenderian S. S. (2020). Neurotoxicity and Cytokine Release Syndrome after Chimeric Antigen Receptor T Cell Therapy: Insights into Mechanisms and Novel Therapies. Front. Immunol. 11, 1973. 10.3389/fimmu.2020.01973 32983132 PMC7485001

[B184] Sim A. J. Jain M. D. Figura N. B. Chavez J. C. Shah B. D. Khimani F. (2019). Radiation Therapy as a Bridging Strategy for CAR T Cell Therapy with Axicabtagene Ciloleucel in Diffuse Large B-Cell Lymphoma. Int. J. Radiat. Oncology*Biology*Physics 105 (5), 1012–1021. 10.1016/j.ijrobp.2019.05.065 PMC6872916 31175906

[B185] Smith C. Økern G. Rehan S. Beagley L. Lee S. K. Aarvak T. (2015). *Ex Vivo* expansion of Human T Cells for Adoptive Immunotherapy Using the Novel Xeno-free CTS Immune Cell Serum Replacement. Clin. Trans. Immunol. 4 (1), e31. 10.1038/cti.2014.31 PMC4318490 25671129

[B186] Smith K. A. (1988). Interleukin-2: Inception, Impact, and Implications. Science 240 (4856), 1169–1176. 10.1126/science.3131876 3131876

[B187] Somerville R. P. Devillier L. Parkhurst M. R. Rosenberg S. A. Dudley M. E. (2012). Clinical Scale Rapid Expansion of Lymphocytes for Adoptive Cell Transfer Therapy in the WAVE Bioreactor. J. Transl. Med. 10, 69. 10.1186/1479-5876-10-69 22475724 PMC3402993

[B188] Sterner R. M. Sakemura R. Cox M. J. Yang N. Khadka R. H. Forsman C. L. (2019). GM-CSF Inhibition Reduces Cytokine Release Syndrome and Neuroinflammation but Enhances CAR-T Cell Function in Xenografts. Blood 133 (7), 697–709. 10.1182/blood-2018-10-881722 30463995 PMC6376281

[B189] Stolp B. Thelen F. Ficht X. Altenburger L. M. Ruef N. Inavalli V. V. G. K. (2020). Salivary Gland Macrophages and Tissue-Resident CD8+ T Cells Cooperate for Homeostatic Organ Surveillance. Sci. Immunol. 5 (46). 10.1126/sciimmunol.aaz4371 32245888

[B190] Stroncek D. F. Reddy O. Highfill S. Panch S. R. (2019). Advances in T-Cell Immunotherapies. Hematology/Oncology Clin. N. Am. 33 (5), 825–837. 10.1016/j.hoc.2019.05.006 31466607

[B191] Tau G. Rothman P. (1999). Biologic Functions of the IFN-Gamma Receptors. Allergy 54 (12), 1233–1251. 10.1034/j.1398-9995.1999.00099.x 10688427 PMC4154595

[B192] Teschner D. Wenzel G. Distler E. Schnürer E. Theobald M. Neurauter A. A. (2011). *In Vitro* stimulation and Expansion of Human Tumour-Reactive CD8+ Cytotoxic T Lymphocytes by Anti-cd3/cd28/cd137 Magnetic Beads. Scand. J. Immunol. 74 (2), 155–164. 10.1111/j.1365-3083.2011.02564.x 21517928

[B193] Tian Y. Li Y. Shao Y. Zhang Y. (2020). Gene Modification Strategies for Next-Generation CAR T Cells against Solid Cancers. J. Hematol. Oncol. 13 (1), 54. 10.1186/s13045-020-00890-6 32423475 PMC7236186

[B194] Tischer S. Priesner C. Heuft H.-G. Goudeva L. Mende W. Barthold M. (2014). Rapid Generation of Clinical-Grade Antiviral T Cells: Selection of Suitable T-Cell Donors and GMP-Compliant Manufacturing of Antiviral T Cells. J. Transl. Med. 12 (1), 336. 10.1186/s12967-014-0336-5 25510656 PMC4335407

[B195] Titov A. Zmievskaya E. Ganeeva I. Valiullina A. Petukhov A. Rakhmatullina A. (2021). Adoptive Immunotherapy beyond CAR T-Cells. Cancers (Basel) 13 (4). 10.3390/cancers13040743 PMC7916861 33670139

[B196] Toprak S. K. (2018). Donor Lymphocyte Infusion in Myeloid Disorders. Transfus. Apher. Sci. 57 (2), 178–186. 10.1016/j.transci.2018.04.018 29754984

[B197] Torres Chavez A. McKenna M. K. Canestrari E. Dann C. T. Ramos C. A. Lulla P. (2019). Expanding CAR T Cells in Human Platelet Lysate Renders T Cells with *In Vivo* Longevity. J. Immunother. cancer 7 (1), 330. 10.1186/s40425-019-0804-9 31779709 PMC6883585

[B198] Torres Chavez A. McKenna M. K. Canestrari E. Dann C. T. Ramos C. A. Lulla P. (2019). Expanding CAR T Cells in Human Platelet Lysate Renders T Cells with *In Vivo* Longevity. J. Immunother. cancer 7 (1), 330. 10.1186/s40425-019-0804-9 31779709 PMC6883585

[B199] Trickett A. E. Kwan Y. L. Cameron B. Dwyer J. M. (2002). *Ex Vivo* expansion of Functional T Lymphocytes from HIV-Infected Individuals. J. Immunol. 262. 10.1016/s0022-1759(02)00018-2 11983220

[B200] Turtle C. J. Hanafi L. A. Berger C. Hudecek M. Pender B. Robinson E. (2016). Immunotherapy of Non-hodgkin's Lymphoma with a Defined Ratio of CD8+ and CD4+ CD19-specific Chimeric Antigen Receptor-Modified T Cells. Sci. Transl. Med. 8 (355), 355ra116. 10.1126/scitranslmed.aaf8621 PMC5045301 27605551

[B201] Turtle C. J. Hanafi L.-A. Berger C. Gooley T. A. Cherian S. Hudecek M. (2016). CD19 CAR-T Cells of Defined CD4+:CD8+ Composition in Adult B Cell ALL Patients. J. Clin. Investig. 126 (6), 2123–2138. 10.1172/jci85309 27111235 PMC4887159

[B202] Turtle C. J. Hay K. A. Hanafi L.-A. Li D. Cherian S. Chen X. (2017). Durable Molecular Remissions in Chronic Lymphocytic Leukemia Treated with CD19-specific Chimeric Antigen Receptor-Modified T Cells after Failure of Ibrutinib. Jco 35 (26), 3010–3020. 10.1200/jco.2017.72.8519 PMC5590803 28715249

[B203] Valton J. Guyot V. Marechal A. Filhol J.-M. Juillerat A. Duclert A. (2015). A Multidrug-Resistant Engineered CAR T Cell for Allogeneic Combination Immunotherapy. Mol. Ther. 23 (9), 1507–1518. 10.1038/mt.2015.104 26061646 PMC4817890

[B204] van der Stegen S. J. C. Hamieh M. Sadelain M. (2015). The Pharmacology of Second-Generation Chimeric Antigen Receptors. Nat. Rev. Drug Discov. 14 (7), 499–509. 10.1038/nrd4597 26129802 PMC6410718

[B205] Viola A. Molon B. Contento R. L. (2008). Chemokines: Coded Messages for T-Cell Missions. Front. Biosci. 1, 6341–6353. 10.2741/3158 18508664

[B206] Vormittag P. Gunn R. Ghorashian S. Veraitch F. S. (2018). A Guide to Manufacturing CAR T Cell Therapies. Curr. Opin. Biotechnol. 53, 164–181. 10.1016/j.copbio.2018.01.025 29462761

[B207] Vucinic V. Quaiser A. Lückemeier P. Fricke S. Platzbecker U. Koehl U. (2021). Production and Application of CAR T Cells: Current and Future Role of Europe. Front. Med. 8, 713401. 10.3389/fmed.2021.713401 PMC8418055 34490302

[B208] Waldman A. D. Fritz J. M. Lenardo M. J. (2020). A Guide to Cancer Immunotherapy: from T Cell Basic Science to Clinical Practice. Nat. Rev. Immunol. 20 (11), 651–668. 10.1038/s41577-020-0306-5 32433532 PMC7238960

[B209] Wallstabe L. Göttlich C. Nelke L. C. Kühnemundt J. Schwarz T. Nerreter T. (2019). ROR1-CAR T Cells Are Effective against Lung and Breast Cancer in Advanced Microphysiologic 3D Tumor Models. JCI Insight 4 (18). 10.1172/jci.insight.126345 PMC6795380 31415244

[B210] Wang M. Munoz J. Goy A. Locke F. L. Jacobson C. A. Hill B. T. (2020). KTE-X19 CAR T-Cell Therapy in Relapsed or Refractory Mantle-Cell Lymphoma. N. Engl. J. Med. 382 (14), 1331–1342. 10.1056/nejmoa1914347 32242358 PMC7731441

[B211] Wang X. Rivière I. (2016). Clinical Manufacturing of CAR T Cells: Foundation of a Promising Therapy. Mol. Ther. - Oncolytics 3, 16015. 10.1038/mto.2016.15 27347557 PMC4909095

[B212] Wang Z. Han W. (2018). Biomarkers of Cytokine Release Syndrome and Neurotoxicity Related to CAR-T Cell Therapy. Biomark. Res. 6 (1), 4. 10.1186/s40364-018-0116-0 29387417 PMC5778792

[B213] Wang Z. Han W. (2018). Biomarkers of Cytokine Release Syndrome and Neurotoxicity Related to CAR-T Cell Therapy. Biomark. Res. 6 (1), 4. 10.1186/s40364-018-0116-0 29387417 PMC5778792

[B214] Watanabe N. Bajgain P. Sukumaran S. Ansari S. Heslop H. E. Rooney C. M. (2016). Fine-tuning the CAR Spacer Improves T-Cell Potency. Oncoimmunology 5 (12), e1253656. 10.1080/2162402x.2016.1253656 28180032 PMC5214260

[B215] Watson H. A. Durairaj R. R. P. Ohme J. Alatsatianos M. Almutairi H. Mohammed R. N. (2019). L-selectin Enhanced T Cells Improve the Efficacy of Cancer Immunotherapy. Front. Immunol. 10, 1321. 10.3389/fimmu.2019.01321 31249570 PMC6582763

[B216] Weber E. W. Parker K. R. Sotillo E. Lynn R. C. Anbunathan H. Lattin J. (2021). Transient Rest Restores Functionality in Exhausted CAR-T Cells through Epigenetic Remodeling. Science 372 (6537), 372. 10.1126/science.aba1786 PMC8049103 33795428

[B217] Wehrli M. Gallagher K. Chen Y. B. Leick M. B. McAfee S. L. El-Jawahri A. R. (2022). Single-center Experience Using Anakinra for Steroid-Refractory Immune Effector Cell-Associated Neurotoxicity Syndrome (ICANS). J. Immunother. Cancer 10 (1). 10.1136/jitc-2021-003847 PMC8744112 34996813

[B218] Westin J. R. Kersten M. J. Salles G. Abramson J. S. Schuster S. J. Locke F. L. (2021). Efficacy and Safety of CD19 ‐directed CAR‐T Cell Therapies in Patients with Relapsed/refractory Aggressive B‐cell Lymphomas: Observations from the JULIET , ZUMA ‐1, and TRANSCEND Trials. Am. J Hematol 96 (10), 1295–1312. 10.1002/ajh.26301 34310745 PMC9290945

[B219] Whilding L. M. Halim L. Draper B. Parente-Pereira A. C. Zabinski T. Davies D. M. (2019). CAR T-Cells Targeting the Integrin αvβ6 and Co-expressing the Chemokine Receptor CXCR2 Demonstrate Enhanced Homing and Efficacy against Several Solid Malignancies. Cancers (Basel) 11 (5). 10.3390/cancers11050674 PMC6563120 31091832

[B220] Wu C. Y. Roybal K. T. Puchner E. M. Onuffer J. Lim W. A. (2015). Remote Control of Therapeutic T Cells through a Small Molecule-Gated Chimeric Receptor. Science 350 (6258), aab4077. 10.1126/science.aab4077 26405231 PMC4721629

[B221] Xu N. Yang X.-F. Xue S.-L. Tan J.-W. Li M.-H. Ye J. (2022). Ruxolitinib Reduces Severe CRS Response by Suspending CAR-T Cell Function Instead of Damaging CAR-T Cells. Biochem. Biophysical Res. Commun. 595, 54–61. 10.1016/j.bbrc.2022.01.070 35101664

[B222] Xu Q. Harto H. Berahovich R. Xu S. Zhou H. Golubovskaya V. (2019). Generation of CAR-T Cells for Cancer Immunotherapy. Methods Mol. Biol. 1884, 349–360. 10.1007/978-1-4939-8885-3_24 30465215

[B223] Xu Y. Chaudhury A. Zhang M. Savoldo B. Metelitsa L. S. Rodgers J. (2016). Glycolysis Determines Dichotomous Regulation of T Cell Subsets in Hypoxia. J. Clin. Investig. 126 (7), 2678–2688. 10.1172/jci85834 27294526 PMC4922684

[B224] Yamada K. M. Sixt M. (2019). Mechanisms of 3D Cell Migration. Nat. Rev. Mol. Cell. Biol. 20 (12), 738–752. 10.1038/s41580-019-0172-9 31582855

[B225] Yáñez L. Sánchez-Escamilla M. Perales M-A. (2019). CAR T Cell Toxicity: Current Management and Future Directions. Hemasphere 3 (2), e186. 31723825 10.1097/HS9.0000000000000186 PMC6746032

[B226] Yang W. W. Bai Y. Xiong S. Meng X. WangLi J. Yan Xu. (2016). Potentiating the Antitumour Response of CD8+ T Cells by Modulating Cholesterol Metabolism. Nature 531 (7596), 651–655. 10.1038/nature17412 26982734 PMC4851431

[B227] Zhang J.-P. Zhang R. Tsao S.-T. Liu Y.-C. Chen X. Lu D.-P. (2018). Sequential Allogeneic and Autologous CAR-T-Cell Therapy to Treat an Immune-Compromised Leukemic Patient. Blood Adv. 2 (14), 1691–1695. 10.1182/bloodadvances.2018017004 30026294 PMC6058233

[B228] Zhang L. Wang S. Xu J. Zhang R. Zhu H. Wu Y. (2021). Etanercept as a New Therapeutic Option for Cytokine Release Syndrome Following Chimeric Antigen Receptor T Cell Therapy. Exp. Hematol. Oncol. 10 (1), 16. 10.1186/s40164-021-00209-2 33608054 PMC7893957

[B229] Zhou J. Jin L. Wang F. Zhang Y. Liu B. Zhao T. (2019). Chimeric Antigen Receptor T (CAR-T) Cells Expanded with IL-7/IL-15 Mediate Superior Antitumor Effects. Protein Cell. 10 (10), 764–769. 10.1007/s13238-019-0643-y 31250350 PMC6776495

[B230] Zhou W. Chen W. Wan X. Luo C. Du X. Li X. (2021). Benefits of Chimeric Antigen Receptor T-Cell Therapy for B-Cell Lymphoma. Front. Genet. 12, 815679. 10.3389/fgene.2021.815679 35126471 PMC8811184

[B231] Zi F. M. Ye L. L. Zheng J. F. Cheng J. Wang Q. M. (2021). Using JAK Inhibitor to Treat Cytokine Release Syndrome Developed after Chimeric Antigen Receptor T Cell Therapy for Patients with Refractory Acute Lymphoblastic Leukemia. Med. Baltim. 100 (19), e25786. 10.1097/md.0000000000025786 PMC8133263 34106613

